# Integrating Diverse Datasets Improves Developmental Enhancer Prediction

**DOI:** 10.1371/journal.pcbi.1003677

**Published:** 2014-06-26

**Authors:** Genevieve D. Erwin, Nir Oksenberg, Rebecca M. Truty, Dennis Kostka, Karl K. Murphy, Nadav Ahituv, Katherine S. Pollard, John A. Capra

**Affiliations:** 1 Gladstone Institute of Cardiovascular Disease, San Francisco, California, United States of America; 2 Institute for Human Genetics, University of California San Francisco, San Francisco, California, United States of America; 3 Department of Bioengineering and Therapeutic Sciences, University of California San Francisco, San Francisco, California, United States of America; 4 Department of Developmental Biology and Department of Computational and Systems Biology, University of Pittsburgh, Pittsburgh, Pennsylvania, United States of America; 5 Department of Epidemiology and Biostatistics, University of California San Francisco, San Francisco, California, United States of America; 6 Center for Human Genetics Research and Department of Biomedical Informatics, Vanderbilt University, Nashville, Tennessee, United States of America; Duke University, United States of America

## Abstract

Gene-regulatory enhancers have been identified using various approaches, including evolutionary conservation, regulatory protein binding, chromatin modifications, and DNA sequence motifs. To integrate these different approaches, we developed EnhancerFinder, a two-step method for distinguishing developmental enhancers from the genomic background and then predicting their tissue specificity. EnhancerFinder uses a multiple kernel learning approach to integrate DNA sequence motifs, evolutionary patterns, and diverse functional genomics datasets from a variety of cell types. In contrast with prediction approaches that define enhancers based on histone marks or p300 sites from a single cell line, we trained EnhancerFinder on hundreds of experimentally verified human developmental enhancers from the VISTA Enhancer Browser. We comprehensively evaluated EnhancerFinder using cross validation and found that our integrative method improves the identification of enhancers over approaches that consider a single type of data, such as sequence motifs, evolutionary conservation, or the binding of enhancer-associated proteins. We find that VISTA enhancers active in embryonic heart are easier to identify than enhancers active in several other embryonic tissues, likely due to their uniquely high GC content. We applied EnhancerFinder to the entire human genome and predicted 84,301 developmental enhancers and their tissue specificity. These predictions provide specific functional annotations for large amounts of human non-coding DNA, and are significantly enriched near genes with annotated roles in their predicted tissues and lead SNPs from genome-wide association studies. We demonstrate the utility of EnhancerFinder predictions through *in vivo* validation of novel embryonic gene regulatory enhancers from three developmental transcription factor loci. Our genome-wide developmental enhancer predictions are freely available as a UCSC Genome Browser track, which we hope will enable researchers to further investigate questions in developmental biology.

## Author Summary

The human genome contains an immense amount of non-protein-coding DNA with unknown function. Some of this DNA regulates when, where, and at what levels genes are active during development. Enhancers, one type of regulatory element, are short stretches of DNA that can act as “switches” to turn a gene on or off at specific times in specific cells or tissues. Understanding where in the genome enhancers are located can provide insight into the genetic basis of development and disease. Enhancers are hard to identify, but clues about their locations are found in different types of data including DNA sequence, evolutionary history, and where proteins bind to DNA. Here, we introduce a new tool, called EnhancerFinder, which combines these data to predict the location and activity of enhancers during embryonic development. We trained EnhancerFinder on a large set of functionally validated human enhancers, and it proved to be very accurate. We used EnhancerFinder to predict tens of thousands of enhancers in the human genome and validated several of the predictions near three important developmental genes in mouse or zebrafish. EnhancerFinder's predictions will be useful in understanding functional regions hidden in the vast amounts of human non-coding DNA.

## Introduction

Eukaryotic gene expression is regulated by a highly orchestrated network of events, including the binding of regulatory proteins to DNA, chemical modifications to DNA and nucleosomes, recruitment of the transcriptional machinery, splicing, and post-transcriptional modifications. Enhancers are genomic regions that influence the timing, amplitude, and tissue specificity of gene expression through the binding of transcription factors and co-factors that increase transcription (as reviewed in [1], [2]). In humans, genetic variation in enhancer regions is implicated in a wide variety of developmental disorders, diseases, and adverse responses to treatments [3], [4], [5].

Enhancers have been discovered in introns, exons, intergenic regions megabases away from their target genes [6], and even on different chromosomes [7]. An enhancer frequently drives only one of many domains of a gene's expression [8], [9] and different cell types accordingly exhibit considerable differences in their active enhancers [10], [11]. This modularity enables the creation of complex regulatory programs that can evolve relatively easily between closely related species [12], [13].

Individual enhancers were initially identified using transgenic assays in cultured cell lines [14], [15] and later *in vivo* in model organisms, such as mouse, *Drosophila*, and zebrafish. In the *in vivo* experiments, a construct containing the sequence to be tested for enhancer activity, a minimal promoter, and a reporter gene (e.g., lacZ) is injected into fertilized eggs, and transgenic individuals are assayed for reporter gene expression.

Early efforts to find enhancers at the genome scale used comparative genomics. Several studies assayed non-coding regions conserved across diverse species for enhancer activity [16], [17], [18], since functional non-coding regions likely evolve under negative selection. This approach identified many enhancers at a range of levels of evolutionary conservation [19], [20], [21]. However, relying on evolutionary conservation alone has several shortcomings: many characterized enhancers are not conserved between species [22], non-coding conservation is not specific to enhancer elements, and evolutionary patterns provide little information about the tissue and timing of enhancer activity.

Enhancer prediction has been revolutionized by recent technological advances, including chromatin immunoprecipitation coupled with high-throughput sequencing (ChIP-seq) [23], RNA sequencing (RNA-seq), and sequencing of DNaseI-digested chromatin (DNase-seq) [24] or formaldehyde-assisted isolation of regulatory elements (FAIRE-seq) [25]. These “functional genomics” assays enable genome-wide measurement of histone modifications, binding sites of regulatory proteins, transcription levels, and the structural conformation of DNA. The ENCODE project [26], FANTOM project [27], and similar studies focused on specific cell types [28], [29] have dramatically increased the amount of publicly available functional genomics data.

Functional genomics studies revealed several genomic signatures of active enhancers. For example, known enhancers are associated with the unstable histone variants H3.3 and H2A.Z [30], [31] and low nucleosome occupancy [32], although these chromatin states are not unique to enhancers. Monomethylation of lysine 4 on histone H3 (H3K4me1), a lack of trimethylation at the same site (H3K4me3), and acetylation of lysine 27 on histone H3 (H3K27ac) may distinguish active enhancers from promoters [10], [33], [34], enhancers that are “poised” for activity later in development [35], [36], and regulatory elements that repress gene expression [37], [38]. Additional features that pinpoint specific classes of active enhancers include binding of the transcriptional cofactor p300/CBP [18], [39], [40], [41], clusters of transcription factor (TF) binding sites [42], [43], [44], [45], and enhancer RNA transcription (eRNAs) [46]. Collectively, functional genomics data have pinpointed the locations of many novel enhancers and yielded insights into sequence and structural determinants of enhancer activity. However, these patterns have not proven to be universal [47], [48], and there is unlikely to be a single chromatin signature that identifies all classes of enhancers [11], [49], [50].

Given the complexity of these functional genomics data sets, computational methods have been developed to improve and generalize the enhancer predictions made from simple combinations of these data. Support vector machines (SVMs) and linear regression models trained to interpret DNA sequence motifs underlying known enhancers have successfully identified novel enhancers active in heart [51], hindbrain [52], and muscle [53] development. Another approach used SVMs to learn patterns of short DNA sequence motifs that distinguish markers of potential enhancers, such as p300 and H3K4me1, in different cellular contexts [54], [55]. Random forests have been used to predict p300 binding sites from histone modifications in human embryonic stem cells and lung fibroblasts [56]. Machine-learning algorithms have also been applied to the related problem of selecting functional TF binding sites out of the thousands of hits to a TF's binding motif throughout the genome [57], [58], [59], [60], [61], [62], [63]. Finally, two groups have taken a less supervised approach and used hidden Markov models (ChromHMM) [64] and dynamic Bayesian networks (Segway) [65] to segment the human genome into regions with unique signatures in ENCODE data and then assigned potential functions, such as enhancer activity, to these states.

While rich datasets coupled with sophisticated algorithms have successfully identified many novel enhancers, comprehensive enhancer prediction is challenging for two main reasons. First, no single type of data is currently sufficient to identify all enhancers active in a given context. Many of the approaches described above use a single mark or motif as a proxy for an enhancer, but this gives an incomplete representation of all biologically active enhancers. Second, while a great deal of functional genomics data are available for different cell lines and tissues, it is not understood how informative experiments in a given cellular context are indicative of enhancer activity in other contexts.

With these issues in mind, we introduce EnhancerFinder, a new two-step machine-learning method for predicting enhancers and their tissue specificity. In machine learning, a classification algorithm is trained to distinguish between labeled training examples (e.g., enhancers and non-enhancers) based on features of these labeled examples (e.g., evolutionary conservation, chromatin signature, DNA sequence). The trained classifier can then be used to predict the labels for uncharacterized genomic regions (e.g., which ones are enhancers). Our approach employs two rounds of a supervised machine-learning technique called multiple kernel learning (MKL) [66], [67]. MKL is based on the theory of SVMs [68], but provides greater flexibility to combine diverse data (e.g., evolutionary conservation, sequence motifs, and functional genomics data from different cellular contexts) and to interpret their relative contributions to the resulting predictions. Our implementation of EnhancerFinder applies MKL in two steps with the goal of generating a genome-wide set of developmental enhancers to better characterize gene regulation during development. The algorithm, which is trained using *in vivo* validated enhancers from the VISTA enhancer database [69] and publicly available genomic data, first aims to distinguish human developmental enhancers from the genomic background and then in a second step predicts enhancer tissue specificity. In contrast to most other enhancer prediction strategies, which are trained on epigenetic marks or sequence motifs that serve as a proxy for a subset of all active enhancers, our use of a heterogeneous and *in vivo* validated set of enhancers, enables us to investigate the complex suite of features that underlie active regulatory regions. With appropriate training data, EnhancerFinder could be applied to study gene regulation at other developmental stages.

Our analyses demonstrate that EnhancerFinder's integration of diverse types of data from different cellular contexts significantly improves prediction of validated enhancers over approaches based on a single context or type of data. We find that enhancers active in some developmental contexts are easier to identify than others. Applying EnhancerFinder to the entire human genome allowed us to predict more than 80,000 developmental enhancers, with tissue-specific predictions for brain, limb, and heart. These predictions significantly overlap known non-coding regulatory regions and are enriched near relevant genome-wide association study (GWAS) lead single nucleotide polymorphisms (SNPs) and genes expressed in the predicted tissue. To illustrate the utility and accuracy of our genome-wide enhancer predictions, we used them to investigate the enhancer landscape near three developmentally expressed genes. First, we screened predicted enhancers near *FOXC1* and *FOXC2* in transgenic zebrafish, and found that 70% (7 of 10) of tested EnhancerFinder predictions have confirmed (6) or suggestive (1) developmental enhancer activity. In addition, we validated a novel cranial nerve enhancer near the *ZEB2* locus using a transgenic mouse enhancer assay. Taken together, our results suggest that the EnhancerFinder approach of integrating diverse data sets significantly improves prediction of biologically active enhancers, providing high-confidence candidate enhancers for studies in developmental gene regulation.

## Results

**Figure 1:**
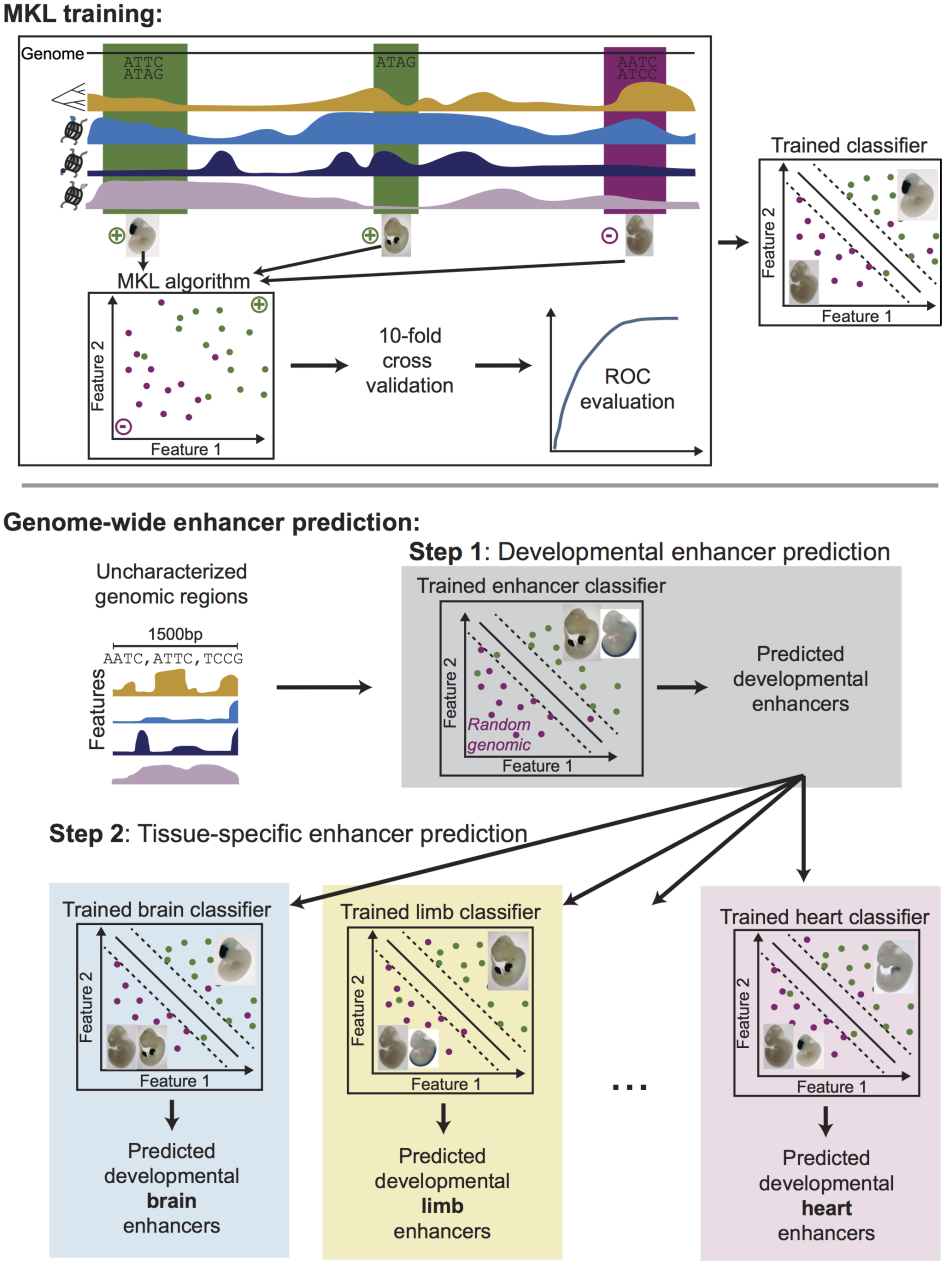
Overview of the EnhancerFinder enhancer prediction pipeline. In our two-step approach, regions of the genome are characterized by diverse features, such as their evolutionary conservation, regulatory protein binding, chromatin modifications, and DNA sequence patterns. For each step, appropriate positive (green) and negative (purple) training examples are provided as input to a multiple kernel learning (MKL) algorithm that produces a trained classifier. We used 10-fold cross validation to evaluate the performance of all classifiers. In Step 1, we trained a classifier to distinguish between known developmental enhancers from VISTA and the genomic background. In Step 2, we trained several classifiers to distinguish enhancers active in tissues of interest from those without activity in the tissue according to VISTA. We applied the trained enhancer classifier from Step 1 to the entire human genome to produce more than 80,000 developmental enhancer predictions. We then applied the tissue-specific enhancer classifiers from Step 2 to further refine our predictions.

We present EnhancerFinder, a machine learning-based enhancer prediction pipeline that allows the seamless integration of feature data from a variety of experimental techniques and biological contexts that have previously been used individually to predict enhancers (Figure 1). We use MKL to integrate these data. MKL algorithms learn a weighted combination of different “kernel” functions that quantify the similarity of different feature data in order to make predictions. In EnhancerFinder, we use three kernels based on different types of biological feature data: DNA sequence motifs, evolutionary conservation patterns, and functional genomics datasets.

EnhancerFinder could be used to predict enhancers active at any stage and tissue. In this study, we evaluate EnhancerFinder's ability to predict developmental enhancers and their tissue specificity.

### A two-step approach to tissue-specific enhancer prediction

Step 1 of our pipeline aims to distinguish all enhancers active in the context of interest (e.g., a specific developmental stage) from non-enhancer regions. Step 2 then builds classifiers to predict the tissues in which the enhancer candidates from Step 1 are active. This two-step approach allows us to accurately identify enhancers, while also distinguishing their tissues of activity.

We train and evaluate EnhancerFinder using the VISTA Enhancer Browser, which at the time of our analysis contained over 700 human sequences with experimentally validated enhancer activity in at least one tissue at embryonic day 11.5 (E11.5) in transgenic mouse embryos. VISTA also contained a similar number of regions without enhancer activity in this context. E11.5 in mouse development roughly corresponds to E41 (Carnegie stage 17 [70]) in human development. In Step 1 of EnhancerFinder, we used all 711 VISTA enhancers as positive training data, and for negative training data, we created a set of 711 random regions matched to the length and chromosome distribution of the positives to represent the genomic background. We did not use the VISTA negatives as negative training examples in Step 1, because they are not representative of all non-enhancer regions (see below). Our goal in Step 1 is to develop a method that can be used to scan the whole genome and distinguish developmental enhancer regions from non-enhancer regions.

The second step of EnhancerFinder aims to distinguish enhancers active in a given embryonic tissue from non-enhancers and enhancers active in other tissues. We consider all enhancers in VISTA with activity in a tissue of interest as positives and all other regions in VISTA (including regions not active at E11.5) as negatives (see Methods). This second step that includes enhancers active in other tissues as negatives in the training proves to be essential for obtaining high specificity in predicting tissue of activity (see below), and it is important to do this in two steps rather attempting to distinguish enhancers of a given tissue from genomic background in one step.

To evaluate EnhancerFinder, we compared it to several commonly used enhancer prediction approaches. Unless otherwise noted, we evaluated the performance of all prediction algorithms using 10-fold cross validation to compute the area under the curve (AUC) for receiver operating characteristic (ROC) curves. We also computed precision-recall curves (Figure S1) and compared power at a low false positive rate.

### Building a general predictor from a biased training set

Because EnhancerFinder learns enhancer signatures from a training data set, we first explored biases in the VISTA enhancers that might affect how well EnhancerFinder could generalize to the whole genome. The genomic regions tested by VISTA were not selected randomly, and thus their positives do not represent a random sample of active enhancers. Nearly all regions tested by VISTA are evolutionarily conserved across mammals (706 of 711 positives and 727 of 736 negatives). Since our goal is to predict a broadly applicable, high confidence set of developmental enhancers, we did not include this feature when making genome wide predictions. However, with this bias in mind, we did evaluate several models that incorporate the degree of evolutionary conservation (see below).

In addition to conservation, several studies deposited in VISTA have considered enhancer-associated proteins and histone marks, such as p300, H3K27ac, and H3K4me1. We collected all data sets of these types from ENCODE and computed their overlap with VISTA enhancers. Fewer than half of the VISTA positives are marked by all three of p300, H3K27ac, and H3K4me1 (from any data set), with substantial percentages marked by only one or two and 13% (93/711) marked by none (Figure S2). These findings indicate that VISTA positives are not highly biased towards a single type of ChIP-seq feature, motivating us to include these features in our genome-wide predictions, with the caveat that the trends we observe for VISTA positives might not generalize to all classes of enhancers. Our analysis also suggests that the standard practice of equating active enhancers with all regions marked by a single ChIP-seq feature, or even the union of overlapping peaks from several ChIP-seq experiments, will fail to identify all active enhancers in a given context.

### EnhancerFinder integrates diverse data types to accurately identify developmental enhancers

**Figure 2:**
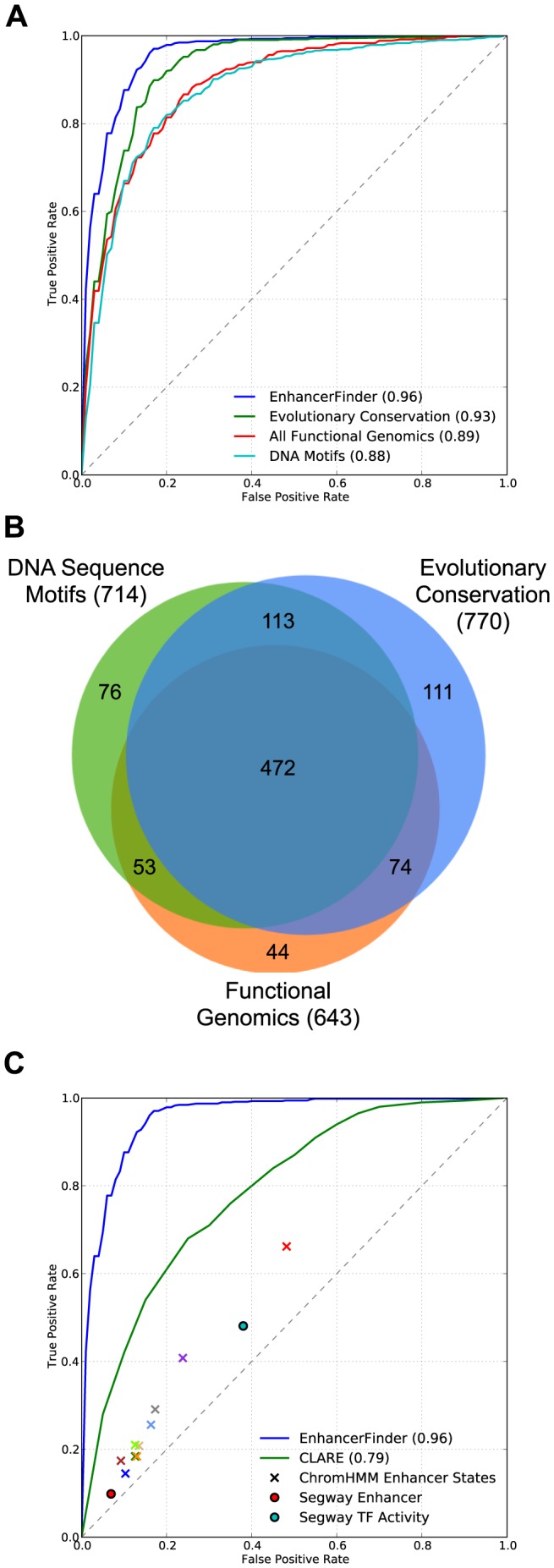
Combining diverse data using EnhancerFinder improves the identification of developmental enhancers. (A) Enhancer prediction strategies based on functional genomics data, evolutionary conservation, and DNA sequence motif patterns all perform well, but EnhancerFinder, which combines these data, provides significant improvement over each of them alone (p<2.0E-7 for all). (B) Each of the approaches from (A) predicts that somewhat different sets of the VISTA regions are enhancers. This suggests that complementary information is contained in each data source. EnhancerFinder (not shown), which combines them, captures many of the enhancers that are unique to each source; it predicts 25 of the 44 enhancers unique to **Functional Genomics**, 30 of the 76 unique to **DNA Sequence Motifs**, and 34 of the 111 unique to **Evolutionary Conservation**. (C) EnhancerFinder outperforms CLARE, a successful enhancer prediction method based on known regulatory motifs. We also evaluated the enhancer states predicted by ChromHMM and Segway, two unsupervised clustering methods that have been used to segment the genome into different functional states based on patterns in functional genomics data, though these methods were not applied to developmental contexts. The different X's represent state predictions based on data from different ENCODE cell types: GM12878 (blue), H1-hESC (violet), HepG2 (brown), HMEC (tan), HSMM (gray), HUVEC (light green), K562 (green), NHEK (orange), NHLF (light blue), and all contexts combined (red).

EnhancerFinder predicts enhancers by integrating classifiers based on distinct data types. In our first evaluation of EnhancerFinder, we consider: functional genomics data, evolutionary conservation patterns, and DNA sequence motifs. Combining these different approaches enables EnhancerFinder to accurately distinguish enhancers from the genomic background (Figure 2A; AUC = 0.96).

The functional genomics component of EnhancerFinder (which we refer to as **All Functional Genomics**) is a linear SVM that incorporates 2469 datasets generated by the ENCODE project and smaller scale studies. These include DNaseI hypersensitivity data and ChIP-Seq for p300, many histone modifications, and many TFs from many adult and embryonic tissues and cell lines (Table S1). DNA sequence patterns are integrated via a 4-spectrum kernel (**DNA Motifs**), which summarizes the occurrence of all length four DNA sequences (4-mers) in input regions [71]. We found that little was gained by increasing *k*, considering multiple *k* simultaneously, or incorporating knowledge of transcription factor binding site (TFBS) motifs as in a previous approach [52] (Figures S3 and S4). Finally, evolutionary conservation information is incorporated with a linear SVM that uses mammalian phastCons scores [72] as features (**Evolutionary Conservation**).

### EnhancerFinder performs significantly better than enhancer prediction approaches based on a single type of data

One motivation for developing EnhancerFinder was to explore whether combining previous successful approaches to enhancer prediction would improve performance. Each of the classifiers combined in EnhancerFinder is representative of a different strategy for predicting enhancers. Thus, we compared the performance of EnhancerFinder to each of its constituents, which are SVMs trained on the same enhancer data as EnhancerFinder, but using only one type of the data features (e.g., only sequence motifs). EnhancerFinder significantly outperformed each of the individual classifiers (Figure 2A; p = 2.0E-7 for **Evolutionary Conservation**, p = 2.6E-8 for **DNA Motifs**, and p = 4.4E-16 for **All Functional Genomics**, McNemar's test), suggesting that these different types of data capture unique aspects of enhancers that are not completely encompassed by any single data type.

Not surprisingly, we found that of the three component classifiers in EnhancerFinder, **Evolutionary Conservation** yields the best performance (AUC = 0.93). As noted above, nearly all regions tested for enhancer activity by VISTA (positives and negatives) are evolutionarily conserved compared to the genomic background. Nonetheless, considering additional features significantly improved predictions. The **DNA Motifs** (AUC = 0.88) and **All Functional Genomics** (AUC = 0.89) classifiers also exhibit strong performance, but also do not perform as well as the combined classifier. EnhancerFinder has nearly twice the power of any of the individual classifiers at a 5% false positive rate (FPR), and its power advantage is even larger at lower FPRs.

**All Functional Genomics**, **DNA Motifs**, and **Evolutionary Conservation** achieve roughly similar performance from different feature data, but each individual classifier predicts a somewhat different set of enhancers during evaluation (Figure 2B). Roughly two-thirds of the enhancer predictions are shared between the three classifiers. The improvement provided by combining these data argues that these data sources are indeed complementary.

We also compared EnhancerFinder's performance with several current computational methods used to identify enhancers. We were able to make the most direct comparison with CLARE, a popular method for identifying enhancers from DNA sequence data, i.e., transcription factor binding site motifs and other sequence patterns [73]. This approach, which has been successfully applied in several contexts [51], [52], [53], [74], makes few assumptions about the input, and is publicly available as a web server. On our Step 1 enhancer prediction task, we find that CLARE achieves an ROC AUC of 0.79. This is much lower than **DNA Motifs** (AUC = 0.88), our approach based on sequence data alone, and the full **EnhancerFinder** (AUC = 0.96; Figure 2C). At a 5% FPR, the power of CLARE is about 20%, compared to approximately 30% for **DNA Motifs** and more than 60% for **EnhancerFinder**.

Comparisons with additional methods were complicated by the fact that most were developed in different contexts. We designed EnhancerFinder specifically to predict biologically active developmental enhancers. Most existing approaches focus on data from a single cell line and define enhancers based on specific enhancer-associated marks or proteins (such as p300 in human embryonic stem cells) rather than biological activity. Thus, we did not anticipate that they would perform as well as EnhancerFinder at developmental enhancer prediction. However, since the predictions of these methods are commonly used outside the specific contexts in which they were made, we believe that it is useful to evaluate how well they can identify developmental enhancers and how much the EnhancerFinder approach applied to developmental enhancers improves on their performance.

In particular, we compared EnhancerFinder to ChromHMM and Segway [64], , two unsupervised machine learning methods for segmenting the genome into a small number of functional “states” based on consistent patterns in ENCODE data for individual cell lines. The states resulting from the segmentations of each cell line's data are annotated by hand into predicted functional classes, which include enhancer activity. To evaluate these methods, we considered the states overlapping our training and testing regions. Any region with an overlapping enhancer state was considered a predicted enhancer and all others were predicted non-enhancers. In this way, we obtained a single point in ROC space for the state predictions. Since there is no score or confidence value associated with the state assignments, a full ROC curve could not be created for these methods. Figure 2C gives the performance for several versions of ChromHMM and Segway based on ENCODE data from different cell lines. Both methods perform better than random, but considerably worse than EnhancerFinder and CLARE (p≈0). We stress that, in contrast to our supervised method, these methods were not explicitly trained to perform the same task as EnhancerFinder, and thus we did not expect them to perform as well as EnhancerFinder. Indeed, these results argue that their utility in identifying developmental enhancers is limited compared to specialized approaches.

### Integrating diverse functional genomics data improves enhancer prediction

As illustrated above, our machine learning prediction and evaluation framework enabled us to quantitatively explore the utility of different genomics datasets in enhancer prediction by creating classifiers based on different types of data (i.e., sequence motifs, evolutionary conservation, and functional genomics) and comparing their performance. We also used this framework to investigate other questions about the utility of different subsets of these data for enhancer prediction. For example, one might expect that some of the datasets included in **All Functional Genomics** (e.g., experiments in cancer cell lines or adult tissues) would not be as useful as others (e.g., experiments in embryonic tissues) for predicting developmental enhancers, and that limiting the features examined by the classifier to the most relevant experiments might improve performance.

To explore this hypothesis, we trained linear SVM classifiers to predict VISTA enhancers (as in Step 1 of EnhancerFinder) based on different subsets of all the functional genomics features (Table 1) and compared their performance. First, we considered a collection of 244 datasets from embryonic tissues and cell lines (**Embryonic Functional Genomics**). Next, we created a classifier that considers data from a wider range of contexts by training a linear SVM using a large, manually curated set of 509 potentially relevant functional genomics data sets (**Relevant Functional Genomics**). This set includes embryonic datasets, along with additional DNaseI and ChIP-seq data from adult tissues and cell lines related to the dominant tissues of activity in VISTA. For example, we included data from human cardiac myocytes, since there are many developmental heart enhancers in our training examples. We compared these to the **All Functional Genomics** classifier described above that uses all 2496 functional genomics features.

**Table 1:** Performance (ROC AUC) of classifiers on each tissue-specific enhancer prediction task (Step 2).

	Heart	Limb	Forebrain	Midbrain	Hindbrain	Neural Tube
Evolutionary Conservation	0.78	0.58	0.52	0.54	0.53	0.52
DNA Motifs	0.83	0.64	0.66	0.63	0.62	0.60
Functional Genomics	0.86	0.74	0.72	0.72	0.69	0.62
Enhancer Finder	0.85	0.74	0.72	0.72	0.69	0.62

**Figure 3:**
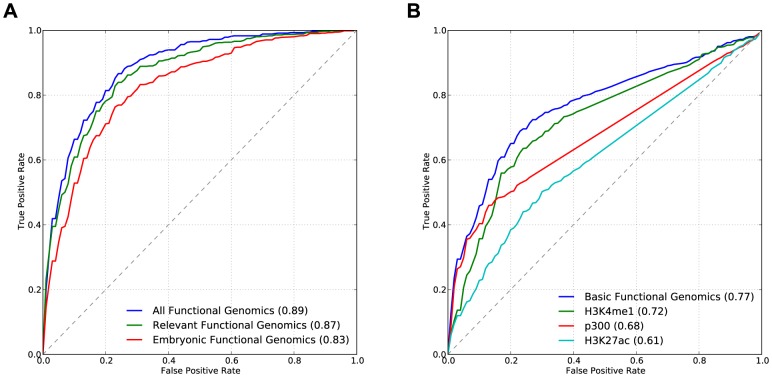
Integrating diverse functional genomics data improves enhancer prediction. (A) Considering functional genomics features from contexts and assays not directly associated with developmental enhancer activity (**All Functional Genomics** and **Relevant Functional Genomics**) improves the identification of developmental enhancers (p = 9.2E-9 and p = 2.7E-6, respectively, compared to **Embryonic Functional Genomics** only). (B) Combining available H3K4me1, p300, and H3K27ac data, which are commonly used in isolation to identify enhancers, in a linear SVM (**Basic Functional Genomics**) is better able to distinguish known developmental enhancers from the genomic background than considering each type of data alone (p<2E-7, for each). However, combining these marks still performs significantly worse than EnhancerFinder (Figure 2A; AUC = 0.96) and considering additional data as in (A).

**All Functional Genomics** (AUC = 0.89) performed slightly, but not significantly, better than **Relevant Functional Genomics** (AUC = 0.87; p = 0.16), and both significantly outperformed **Embryonic Functional Genomics** (AUC = 0.83; p = 9.2E-9 and p = 2.7E-6, respectively) (Figure 3A). At low FPRs, the differences in power between these classifiers were modest. The **Embryonic Functional Genomics** classifier included the most time-appropriate datasets, yet its performance was improved by including additional data sets that seem less relevant to our classification problem *a priori*. Thus, we conclude that it can be advantageous to consider a range of functional genomics features, especially when few features are available from the context of interest. The utility of these additional data sets might indicate that some enhancer features are stable across cell types and developmental stages, but it could also reflect information these data provide about genomic regions that are *not* active enhancers during development (see Discussion).

### Histone marks and p300 provide complementary information about enhancer activity

We also explored the utility of individual functional genomics datasets that are often used as proxies for developmental enhancers by creating three linear SVM classifiers: **H3K27ac**, **H3K4me1**, and **p300**. These SVMs were trained to distinguish VISTA positives from the genomic background (Step 1) using all available data of the specified type from ENCODE, which include a range of cell types and tissues (Table S1). All three classifiers performed better than random (Figure 3B). **H3K4me1** (AUC = 0.72) and **p300** (AUC = 0.68) performed similarly (p = 0.25), with **p300** performing best at low FPRs and **H3K4me1** best at higher FPRs. Both significantly outperformed **H3K27ac** (AUC = 0.61; p = 9.4E-15 and p = 5.5E-9, respectively); however, we caution against extrapolating from this comparison, since it may reflect biases in the feature sets available and the VISTA positives. Since combinations of these features are often used to predict enhancers, we next trained a linear SVM classifier (**Basic Functional Genomics**) that includes all three data types together. The combined classifier significantly outperforms all the individual classifiers (AUC = 0.77; p<2E-7 for each), suggesting that each data type contributes unique information about enhancer activity. Also, all four SVM classifiers achieved much better performance than the common approach of simply considering regions overlapping with these data (Figure S5).

EnhancerFinder also learns weights for individual features within classifiers that reflect their contribution to the enhancer predictions. We found that features known to be associated with enhancer activity in relevant cellular contexts generally receive positive weights, while those associated with other types of elements received negative weights (Text S1 and Figure S6).

### EnhancerFinder's two-step approach enables tissue-specific enhancer prediction

In the previous sections, we focused on generic developmental enhancer prediction (Step 1 of EnhancerFinder). Step 2 of EnhancerFinder applies a second round of MKL to refine and further annotate predicted enhancers from Step 1 (Figure 1). In this study, Step 2 consists of training an MKL classifier to distinguish VISTA enhancers active in a given tissue from VISTA regions without activity in that tissue, i.e., non-enhancers from VISTA plus enhancers for other tissues. We did not require that the positive training examples be active *only* in the tissue of interest. Using the same feature data as in Step 1, we created tissue-specific classifiers for all tissues with more than 50 examples in VISTA: forebrain, midbrain, hindbrain, heart, limb, and neural tube.

**Figure 4:**
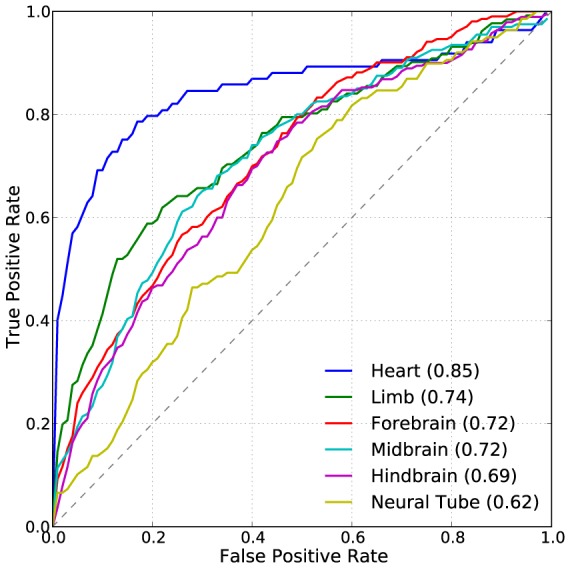
Enhancers of heart expression are easier to identify than enhancers active in other tissues at E11.5. (A) In Step 2 of our prediction pipeline, we trained EnhancerFinder using the same features as in Step 1 (Figure 1), but using VISTA enhancers active in a given tissue as positives and tested regions that did not show activity in the tissue as negatives. Heart enhancers were dramatically easier to distinguish from other enhancers than enhancers of expression in other tissues. The heart enhancers have significantly higher GC content than other enhancers and the genomic background. This and several other unique attributes may explain the ease of identifying them (Figures S7 and S8). In general, functional genomics data are the most informative data type for predicting enhancer tissue specificity (Table 1).

The performance of EnhancerFinder's tissue specificity predictions varied dramatically between tissues (Figure 4), with the best performance for heart (AUC = 0.85), followed by limb (AUC = 0.74), forebrain (AUC = 0.72), midbrain (AUC = 0.72), hindbrain (AUC = 0.69), and neural tube (AUC = 0.62), which was the worst of the tested tissue classifiers, but better than random. We combined all brain enhancers into one class, and the performance of this generic brain classifier was similar to that of the more specific brain classifiers (AUC = 0.73). The EnhancerFinder tissue-specific classifiers trained with all data types performed well for most tissues (Table 1); however, classifiers based on functional genomics alone often performed as well as the full EnhancerFinder classifier, suggesting functional genomics data are more informative about developmental enhancer tissue specificity than degree of conservation or sequence motifs.

**Figure 5:**
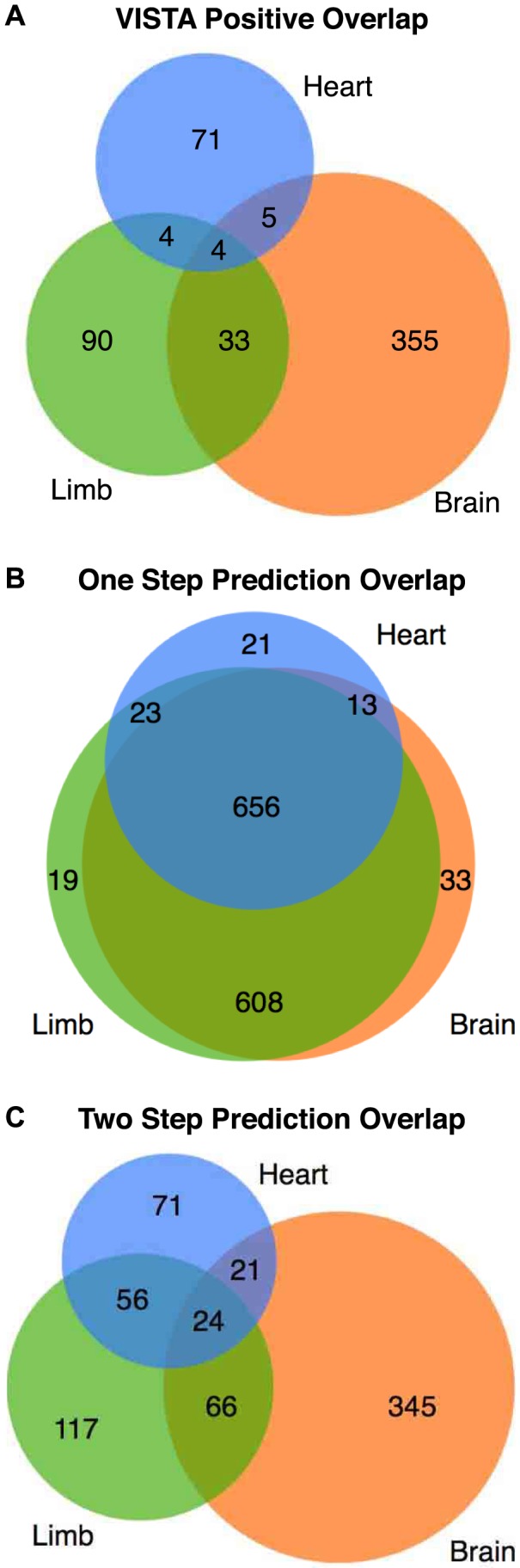
EnhancerFinder's two-step approach captures tissue-specific attributes of enhancers. (A) The true overlap of human enhancers of brain, heart, and limb in the VISTA database. The vast majority of characterized enhancers are unique to one of these tissues at this stage. For example, of the 84 validated heart enhancers, 71 are unique to heart, five are shared with brain, four with limb, and four with both. (B) The predicted overlap of VISTA enhancers based on predictions made with a single training step using MKL with only enhancers of that tissue considered positives and the genomic background as negatives. This approach overestimates the number of enhancers active in multiple tissues. Each classifier mainly learns general attributes of enhancers, rather than tissue-specific attributes. (C) The predicted overlap based on EnhancerFinder's two-step approach. These predictions are much more tissue-specific and exhibit overlaps between tissues similar to the true values (A). Predicted tissue distributions are similar when the methods are applied to other genomic regions, as illustrated in our genome-wide predictions, but only predictions on VISTA enhancers are shown here to enable comparisons to the distribution for validated enhancers (A).

Most previous efforts to predict tissue-specific enhancers have performed a single training step using enhancers or enhancer marks present in the tissue of interest as positives and non-enhancer regions or the genomic background as negatives. To test whether our two-step method improves upon these previous approaches, we trained one-step MKL tissue-specific classifiers and compared their predicted tissue distributions to those of validated enhancers from the VISTA database (Figure 5A). First, we trained a set of tissue-specific classifiers using enhancers active in each tissue as positives and the genomic background as negatives. These classifiers predict very similar sets of enhancers regardless of the target tissue; and they vastly overestimate the number of enhancers that are active in multiple tissues (95% of predictions versus 8% of VISTA) and the number of true enhancers of each tissue (Figure 5B). In contrast, classifiers trained as in Step 2 of EnhancerFinder, i.e., using tissue-specific enhancers as positives and a mix of enhancers active in other tissues and regions with no activity in VISTA as negatives, show much greater tissue-specificity in their predictions (76%) and a similar amount of overlap as among known enhancers (Figure 5C).

### Heart enhancers are easier to identify due to several unique attributes

The relative ease of identifying heart enhancers is likely due to several unique characteristics. Known heart enhancers at E11.5 are more evolutionarily conserved than genomic background, but significantly less conserved than enhancers in other tissues [39], [41]. In addition, we observed that heart enhancers at this developmental stage are uniquely close to the nearest transcription start site (TSS) (Figure S7). These two patterns are consistent with a recent study of mouse enhancers from different developmental stages [75]. Finally, we observed that E11.5 heart enhancers have an unusually high GC content (49%) compared to enhancers of other tissues at E11.5 (∼40%). A simple classifier based solely on the GC content of a region performs nearly as well as our full classifier for heart enhancers (Figure S8). In contrast, sequence-based classifiers do not perform well on the other tissues whose enhancer GC content is not significantly different from the genomic background (Table 1). The high GC content of heart enhancers is not due to overlap with CpG islands. Only about 4% of VISTA enhancers overlap with a CpG island, and this number is consistent across tissues. We also did not find enrichment for any known GC-rich transcription factor binding site motifs in VISTA heart enhancers. We do see, however, that repeat regions in heart enhancers are depleted for the very AT-rich repeats seen in other enhancers, and that most of the repeat regions in heart enhancers are 40–60% GC. Our results suggest the possible existence of unknown GC-rich motifs that may be important for gene regulation in the cardiac lineage.

The heart classifier based on functional genomics data alone exhibits strong performance compared to other tissue-specific classifiers as well (Table 1). It is possible that this is due to the presence of feature data from contexts more relevant to developmental heart activity than to other tissues, rather than unique attributes of the heart enhancers themselves. Indeed, the highest weighted features in the heart functional genomics classifier come from heart tissues. However, the performance of the heart classifier based only on functional genomics data does not decrease substantially when we exclude data from the most relevant contexts: embryonic heart tissue, adult hearts, and stages of a directed differentiation of stem cells into cardiomyocytes (ROC AUC = 0.85). Thus, it is possible that feature data from less obviously relevant contexts are more informative about heart activity than for other tissues. We suspect that the ease of distinguishing heart enhancers may be due to the earlier development of the heart compared to other tissues (see Discussion).

### We predict more than 80,000 developmental enhancers across the human genome

One of the main motivations for developing algorithms that can distinguish active enhancers is to apply them to unannotated genomic regions to aid the exploration and interpretation of the gene regulatory landscape of the human genome (Figure 1). To produce a genome-wide set of candidate developmental enhancers, we divided the genome into 1.5 kb blocks overlapping one another by 500 bp and applied Step 1 of EnhancerFinder to each of these regions. EnhancerFinder produces a score for each region; positive scores indicate membership in the positive set (enhancers), and negative scores indicate membership in the negative set (non-enhancers). To focus on high confidence predictions in this genome-wide analysis, we used the cross-validation-based evaluation described above to find a 5% FPR score threshold, and only considered regions exceeding this threshold. After merging overlapping positive predictions, we identified 84,301 developmental enhancers across the human genome with median length of 1,500 bp and total genome coverage of 183,695,500 bp (5.86%).

The 5% FPR threshold we used corresponds to a 65% true positive rate (TPR). To calculate the false discovery rate (FDR), we must estimate the unknown fraction of 1.5 kb blocks of the human genome that harbor developmental enhancer regions. If this fraction were as high as 50%, a 5% FPR would correspond to a 9% FDR. If instead we estimate that 10% of 1.5 kb windows contain a developmental enhancer, we see an FDR of 47% at a 5% FPR. While this may seem high, our recent analysis of predicted enhancers with human-specific substitution rate acceleration found a lower failure rate at E11.5 (17%, 5/29) [74], and only three of ten tested predictions did not validate with confirmed or suggestive activity in our zebrafish assay (see below). This suggests that the FDR may be lower in experimental applications, especially when predicted enhancer regions are analyzed in the context of other relevant data. However, to accurately measure the true FDR would require experimental testing of a very large, random set of EnhancerFinder predictions, which is beyond the scope of this study.

In our genome-wide analysis, we used the smaller **Relevant Functional Genomics** data set in order to reduce the computational time required. We also did not include evolutionary conservation data, because the positives in our training data are almost universally conserved. While most enhancers likely exhibit some evolutionary conservation, this extremely high fraction is likely due to bias in the selection of the tested regions in VISTA and could reduce our ability to detect less highly conserved novel enhancers genome-wide (see Discussion). The resulting conservation-free classifier still performed extremely well in cross validation (AUC = 0.92). Supporting this approach, non-conserved regions make up over 20% of our genome-wide enhancer predictions. As noted above, we did not observe any other dramatic biases in the feature data associated with human VISTA enhancers.

**Figure 6:**
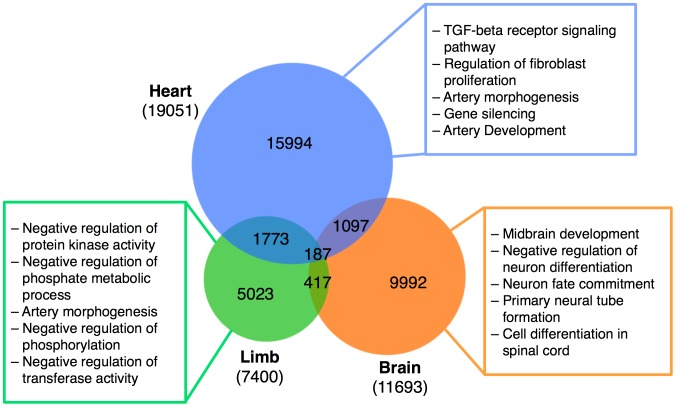
Predicted tissue-specific enhancers exhibit tissue-specific characteristics. EnhancerFinder identifies thousands of novel high-confidence (FPR<0.05) heart, brain, and limb enhancers. These enhancers are enriched for tissue-specific GO Biological Processes. The five most enriched GO Biological Processes among genes near each enhancer set (as calculated using GREAT) are listed in the colored boxes. Nearly 90% of EnhancerFinder predicted heart, brain, and limb enhancers are unique to a single tissue. The larger number of high-confidence heart enhancers relative to brain and limb enhancers is the result of the superior performance of the heart classifier.

Next, we applied Step 2 of EnhancerFinder to all enhancer regions predicted in Step 1. We focused on brain, limb, and heart, because these tissues are highly represented in VISTA and have been extensively studied in previous analyses of developmental enhancers. We predicted 7,400 limb enhancers, 19,051 heart enhancers, and 11,693 brain enhancers (Figure 6) at a 5% FPR threshold tuned separately for each tissue. Since EnhancerFinder makes predictions for each tissue independently, there are no constraints on the distribution of tissues in the resulting genome-wide predictions. Nonetheless, we find a high level of tissue-specificity; nearly 90% of the limb, heart, and brain enhancers are predicted to be active in just one of the three tissues.

All genome-wide enhancer predictions are available as tracks for import into the UCSC Genome Browser (Data File S1). These lists of high-confidence tissue-specific enhancers should not be viewed as exhaustive; we found thousands of regions with positive, but less significant scores from Step 2 of EnhancerFinder.

### Predicted enhancers are associated with relevant functional genomic regions

To characterize and further validate our genome-wide enhancer predictions, we examined their genomic distribution with respect to several independent indicators of function (details in Text S1). Genes near brain and heart enhancers are enriched for expression in relevant tissues (Tables S2 and S3). Similarly, Gene Ontology (GO) Biological Process enrichment analyses of nearby genes suggest that our predicted developmental enhancers target genes that function in relevant cell types and tissues (Figure 6). The most prevalent transcription factor binding site motifs found in the sequences of predicted enhancers differed between enhancers of different tissues and included many relevant developmental TFs (Table S4). Finally, our predicted enhancers contain 676 lead SNPs associated with significant effects in GWAS (Table S5); this is significantly more than expected at random (permutation p<0.001).

Taken together, these analyses suggest that EnhancerFinder identifies many active regulatory regions that contain functionally relevant variation. Our tissue-specific enhancer predictions give valuable annotations to thousands of non-coding regions of the human genome that had not previously been linked to developmental regulation. For example, thousands of SNPs associated with disease by GWAS are in non-coding regions with limited functional annotations [76]. Our genome-wide enhancer predictions provide a resource for exploring the mechanisms and functional effects of these uncharacterized GWAS hits.

### EnhancerFinder predictions function as enhancers in the developing embryo

To demonstrate that genome-wide EnhancerFinder predictions can facilitate the discovery of functional regulatory elements, we present two case studies in which we identify and validate novel enhancers near genes active during development.

#### EnhancerFinder identifies many novel enhancers near *FOXC1* and *FOXC2*

**Figure 7:**
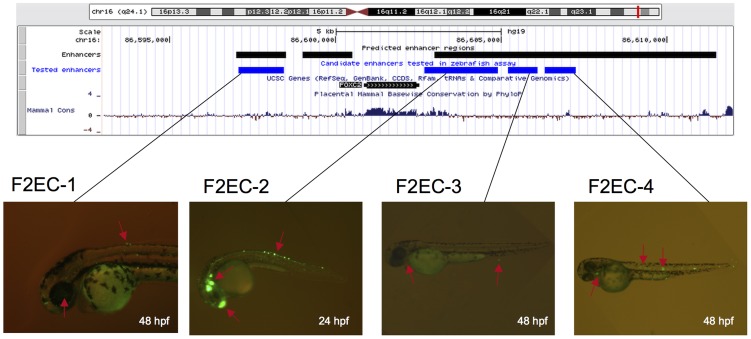
Four novel developmental enhancers near *FOXC2*. This UCSC Genome Browser (http://genome.ucsc.edu) snapshot shows the genomic context of four candidate human enhancers tested in transgenic zebrafish. For each enhancer, we show a zebrafish image that is representative of the reproducible expression patterns. *FOXC2* Enhancer Candidate 1 (F2EC-1) drives expression at 48 hpf in the eye and epidermis (arrows). F2EC-2 shows expression at 24 hpf in the forebrain, midbrain, and nerve. F2EC-3 drives expression at 48 hpf in the epidermis and heart. F2EC-4 shows expression at 48 hpf in the notochord, spinal cord, and heart. See Table S6 for full list of expressed tissues seen in each candidate enhancer and Figure S10 for results on candidate enhancers near *FOXC1*.

To evaluate several EnhancerFinder predictions, we took advantage of a transgenic enhancer assay in embryonic zebrafish (Methods). We tested enhancer activity of ten predicted human enhancers near *FOXC1* and *FOXC2*, two forkhead box TFs. The mouse homologs *Foxc1* and *Foxc2* have been studied extensively and have been shown to be required for proper embryonic development; *Foxc1* null and *Foxc2* null mutants are pre- or perinatal lethal [77], [78], [79]. In humans, complete lack of *FOXC1* is also typically pre- or perinatal lethal, and deletions near and point mutations in *FOXC1* contribute to eye and brain development disorders [80], [81]. Figure 7 shows the genomic context of *FOXC2*, along with the candidate enhancers that we tested (*FOXC2* Enhancer Candidates, or F2ECs). *FOXC1* results are shown in Supplementary Figure S10 (*FOXC1* Enhancer Candidates, or F1ECs). Six of the ten predicted human enhancer sequences showed consistent enhancer activity in zebrafish at 24 or 48 hours post fertilization (hpf) (F1EC-1, F1EC-6, F2EC-1, F2EC-2, F2EC-3, and F2EC-4). One additional candidate enhancer (F1EC-3) showed suggestive enhancer activity. EnhancerFinder predicted tissue specificity for eight of the ten candidate enhancers, and we saw the predicted expression pattern confirmed for just one candidate enhancer (F2EC-3, predicted heart enhancer), and suggestive expression for another (F1EC-6, predicted heart enhancer). However, it is difficult to interpret this result, since the tested stages (24 and 48 hpf) do not directly correspond to single stages of mammalian development, and some of the studied tissues are not homologous. Also, since we tested predicted human enhancer sequences in zebrafish, it is possible that differences in developmental regulation between human and fish contributed to this result.

#### EnhancerFinder predictions highlight a novel enhancer near *ZEB2*

**Figure 8:**
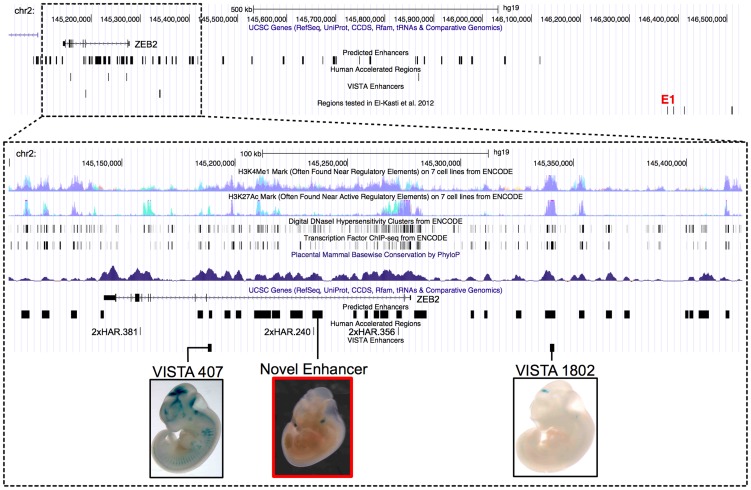
A novel cranial nerve enhancer in the *ZEB2* locus. This UCSC Genome Browser snapshot shows a dense region of predicted enhancers in a 1.5*ZEB2* and part of the adjacent gene desert. Tracks give the locations of four human accelerated regions (HARs), two validated VISTA enhancers (hs407 and hs1802), and the E1 region recently shown to have postnatal enhancer activity [87]. The inset shows a zoomed in view of *ZEB2* (hg19.chr2:145,100,000–145,425,000) along with summaries of several ENCODE functional genomics datasets and evolutionary conservation across placental mammals. We tested the predicted enhancer overlapping 2xHAR.240 for enhancer activity at E11.5 in transgenic mice. Both the human and chimp versions of this sequence drive consistent expression in the cranial nerve (Figure S11).

Next, we sought to investigate a novel enhancer prediction in a mammalian system. We selected the locus containing *ZEB2*, a zinc finger E-box-binding homeobox-2 TF, which has many roles throughout embryonic and postnatal development, in particular in cortical neurogenesis [82], [83], [84], [85]. Mutations in *ZEB2* are associated with Mowat-Wilson syndrome, a complex developmental disorder [86]. However, relatively little is known about the genetic mechanisms that orchestrate *ZEB2*'s expression. A long-range enhancer of postnatal expression in developing kidney cells (E1 in Figure 8) was recently discovered 1.2 megabases (Mb) downstream of *ZEB2* in the adjacent gene desert [87]. Since this enhancer does not fully recapitulate the expression timing and domains of *ZEB2*, the authors speculated that the gene has many other, potentially long-range, enhancers. Supporting this theory, there are two validated E11.5 brain enhancers near *ZEB2* in the VISTA Enhancer Browser (Figure 8, VISTA hs407 and VISTA hs1802). Finally, there is an enrichment of human accelerated regions (HARs) [88], [89] near *ZEB2*, suggesting that it may have human-specific regulatory patterns.

Our EnhancerFinder predictions support the existence of a rich regulatory program specified in the non-coding sequence nearby *ZEB2*; there are 54 predicted enhancers for which it is the nearest TSS. This puts *ZEB2* in the top 0.2% of all genes with respect to the number of adjacent enhancer predictions. Supporting the validity of our predictions, the known VISTA enhancers both overlap EnhancerFinder predicted enhancers, while the regions known to be inactive or active at later postnatal developmental stages (E1) [87] do not

We selected an EnhancerFinder predicted enhancer (indicated in the zoomed pane of Figure 8) for further experimental analysis due to its high EnhancerFinder score and overlap with a HAR (2xHAR.240). We interrogated the potential of the human and chimp sequences at this region to drive gene expression at E11.5 in transient transgenic mouse embryos. All seven embryos with staining showed cranial nerve expression (Figure 8 red box; Figure S11), regardless of whether the construct contained the human or chimp sequence. Thus, we have identified a novel enhancer within the *ZEB2* locus that overlaps one of its expression domains; however, whether this enhancer targets *ZEB2* remains to be proven.

This is not the only HAR enhancer validated to date. In a recent publication, we showed that many HARs function as developmental enhancers [90]. In that study, we experimentally tested 29 HARs that EnhancerFinder predicts to function as developmental enhancers, and found, in agreement with the cross-validation and zebrafish experimental validation rates here, that 24 of the regions (83%) show positive enhancer activity at E11.5. In addition, one EnhancerFinder negative showed no enhancer activity.

While none of the enhancer predictions tested so far were randomly selected, our results suggest that EnhancerFinder is a powerful tool for accurately characterizing developmental regulatory potential in many useful contexts. Our enhancer predictions highlight many additional candidates for further investigation, and we believe that they will enable similar analyses of the regulatory potential of many other genes and regions of interest.

## Discussion

In this study, we developed EnhancerFinder, a new machine-learning framework for predicting regulatory enhancers from diverse data sources. In contrast to most previous enhancer identification strategies, which have based their predictions on one or a small number of data types, EnhancerFinder enables us to flexibly integrate the large and continually expanding collection of evolutionary, DNA sequence, and functional genomics data that are informative about enhancer function. Our analysis of the EnhancerFinder algorithm and its predictions makes three major contributions. First, we demonstrate that integrating diverse types of data from many cellular contexts, including some unexpected ones, can accurately predict *in vivo* validated developmental enhancers. Second, we show that a two-step approach in which enhancer tissue-specificity is individually evaluated after general enhancer prediction improves the identification of enhancers' tissues of activity. Finally, our genome-wide developmental enhancer annotations, including tissue-specific predictions for heart, brain, and limb, assign novel functions in development to thousands of genomic regions. We show that these predictions are enriched for a number of independent indicators of regulatory functions. As a result, we expect our predictions to prove useful in the annotation of non-coding genomic regions, as illustrated in the identification of novel enhancers near *ZEB2*, *FOXC1*, and *FOXC2*. Our genome-wide predictions are freely available as a UCSC Genome Browser track.

### A biologically active *in vivo* definition of “enhancer”

We chose to define developmental enhancers for training as genomic regions that are experimentally shown to activate gene expression *in vivo* in embryonic mouse assays. We believe that this definition is better suited to identifying regions for further exploration and experimental characterization than approaches based on single data sources, such as p300, H3K4me1, or H3K27ac, associated with enhancers in individual cell lines. We showed that our predicted enhancers, based on this biologically active definition, significantly overlap data sets commonly used as proxies for enhancer activity, such as H3K27ac and p300 binding. However, these other data alone are not sufficient to identify all enhancers, as we demonstrated for H3K27ac, H3K4me1, and p300 in Figure 3B. Similarly, when we evaluated the ability of other computational methods to identify enhancers, we find that they perform better than random, but that EnhancerFinder significantly outperforms them at identifying biologically active developmental enhancers. This is not surprising given the different contexts in which some enhancer predictions, such as those from ChromHMM and Segway, were developed.

While EnhancerFinder could be used to predict enhancers in well-characterized cell lines, it is particularly useful at identifying enhancers in complex tissues that contain multiple cell types and in cell types that do not have much specific functional genomics data available. Other computational approaches to enhancer prediction have focused on identifying enhancers in individual cell types using functional genomics data from the same cells [56] or using the differences in cell type specific transcription factor binding to identify cell-type specific binding motifs [61]. These methods generally perform well, but they do not address enhancer prediction in cell types with little or no functional genomics data, or in tissues that contain multiple cell types.

### Why do seemingly irrelevant data improve our enhancer predictions?

Data such as p300 binding sites and H3K4me1 have been used in previous studies to identify enhancers, and these data are major contributors to our enhancer predictions. However, data from other sources and contexts less directly associated with enhancer activity provide complementary information that improves our predictions. Some of these data may be negatively correlated with enhancer activity, allowing EnhancerFinder to learn what features distinguish regions that are not developmental enhancers. Others may help reinforce patterns present in data from more relevant contexts, reflecting some degree of stability in the features of enhancer regions across developmental stages and cell types. For example, we found that features measured in embryonic stem cells are quite useful for E11.5 enhancer prediction; their removal from the classifier degrades performance and/or they have large (positive or negative) MKL weights. Examination of these features suggests that some identify “poised” regions that will become active enhancers upon differentiation, while others seem to help distinguish stem cell enhancers (i.e., non-enhancers at E11.5) from those specific to differentiated lineages. We note that despite these interesting observations, most individual functional genomics features do not carry a great deal of information and the power of EnhancerFinder comes from the integration of different types of data. It is also possible that as a more complete experimental characterization of chromatin state and protein-DNA binding from E11.5 tissues is obtained, data from less relevant contexts will not provide as much improvement as it did in this study.

### What data are most informative about enhancer activity?

We focused on a single developmental stage with a large number of validated enhancers. To efficiently extend enhancer detection and validation to new contexts, it will be very important to select the most informative data to collect. Even though the ENCODE project has produced an impressive amount of data, it still has not extensively assayed most contexts of interest to researchers, in particular developmental biologists. The performance of classifiers trained on subsets of all our data and the weights we learned for feature sets and individual features provide some guidance for future experiments. Evolutionary conservation and DNA sequence patterns are broadly useful in the identification of enhancers, but our results suggest that adding functional genomics data is necessary to make more precise predictions about the contexts of activity. H3K4me1 and p300 are two of the most useful functional genomics data types overall (Figure S6), but many others are useful in particular contexts. However, the non-random sampling of functional genomics data and enhancers makes definitively determining the relative utility of different data types challenging.

### Why are heart enhancers easier to predict than other types of enhancers?

We saw a broad range in our ability to predict the tissue specificity of enhancers from existing data. Heart enhancers were dramatically easier to identify than other tissue-specific enhancers. Heart enhancers have significantly higher GC content than enhancers of other tissues, are less evolutionarily conserved, and are closer to the nearest TSS than other known enhancers at E11.5, and we show that GC content alone is sufficient to accurately predict many heart enhancers (Figures S7Figures S7 and S8). However, functional genomics data alone were also able to accurately predict heart enhancers. The underlying biological explanation for these patterns may have to do with relative developmental age of different organs and tissues. At E11.5, the heart is further along its developmental trajectory than the other tissues considered, and heart enhancers have completed their most conserved developmental stage, whereas forebrain enhancers are most strongly conserved at E11.5 and E14.5 [75]. At E11.5, many of the less conserved, mammal-specific features of the heart are developing [91], [92], whereas other tissues are still developing under more general, less species-specific conserved regulatory programs at E11.5 [93]. A recent study of enhancers in the adult mouse retina found that high local GC content was strongly correlated with enhancer activity [94]. Paired with our result, this suggests that GC content is a distinguishing feature of certain classes of enhancers.

### Limitations of our approach

In spite of the strong overall performance of EnhancerFinder at predicting tissue-specific developmental enhancers, our approach has some limitations. First, we rely heavily upon the VISTA Enhancer Browser for training examples, because it is the largest collection of validated mammalian enhancers currently available. This resource provides an impressive catalog of validated human regulatory enhancers, but it is limited to a single developmental stage and experimental system. Without more data and analysis, it is difficult to evaluate how specific our predictions are to this context. Applying EnhancerFinder to known enhancers in model organisms, such as zebrafish and fly, would provide additional opportunities to evaluate our approach and findings, while potentially demonstrating differences in how enhancers function in these different species.

Second, most of the enhancers present in VISTA are evolutionarily conserved. As a result, the VISTA enhancers cannot be viewed as an exhaustive catalog of the full range of enhancers. However, these regions have validated enhancer activity *in vivo*, and thus provide an appealing alternative to approaches that use single-mark proxies for enhancer activity (e.g., considering all H3K27ac peaks as active enhancer regions). In addition to being conserved, these regions contain many signatures of enhancers in their sequence motifs and functional genomics composition that are useful for predicting enhancers. To emphasize these features and mitigate the impact of bias towards conserved regions, we removed evolutionary conservation as a feature from EnhancerFinder when we applied it to predict enhancers genome-wide. Our goal in doing so was to improve our ability to discern less conserved enhancers in these genome-wide predictions, and indeed, we predicted thousands of non-conserved enhancers (∼20% of all predictions).

Third, though our predictions are based on a large collection of genome-wide chromatin state, protein-binding, and sequence information from many contexts, we are still limited by data availability. Even with the impressive efforts of ENCODE and related projects, producing data that are perfectly matched to all contexts of interest is time consuming and sometimes impossible, especially when studying humans. Thus, it will be important to develop a principled understanding of how different data can be generalized across tissues, developmental stages, and between species. In our analysis, many of the highest weighted features come from contexts close to the developmental stage of interest, and thus we anticipate that gathering more data from developmentally relevant cells and tissues will significantly improve our ability to annotate genomic regions involved in the regulation of embryonic development. However, data from other, seemingly unrelated, contexts may continue to prove useful.

### Extensions and future applications

This study annotates regulatory elements in the human genome and provides tools for interpreting the effects of mutations in non-coding regions. Our case studies on regions around *ZEB2*, *FOXC1*, and *FOXC2* illustrate how our predictions can facilitate the rapid identification of novel enhancers. In addition, the statistical enrichment for GWAS SNPs in our genome-wide enhancer predictions suggests that they may be a good resource for pinpointing causal mutations in potential disease loci.

EnhancerFinder is a general framework for enhancer prediction and evaluation of different data sources that aim to annotate the regulatory functions of the human genome. It could easily be extended to include additional types of data, such as population-level variation at each locus, information about the three-dimensional state of the genome from Hi-C and 5C, and predictions of potential target genes for each enhancer. It could also be used to analyze additional aspects of the data we already consider, such as accounting for the relative genomic position of different features [66].

The EnhancerFinder two-step approach enables delineation of features common to all enhancers versus those that characterize enhancers of different types. For example, we find that predicting enhancers that are unique to a single tissue is more difficult than those that are active in multiple tissues (Figure S9), that certain features make prediction of heart enhancers particularly easy, and that different features are selected in classifiers for general enhancers and those for specific tissues. Together, these results suggest that there may be distinct classes of enhancers, even among those active in a given tissue at a single developmental stage. Further analysis of EnhancerFinder classifiers based on different types of data may help suggest biological mechanisms underlying the functional distinctions and genomic features of these different classes of enhancers.

## Methods

### Ethics statement

Transgenic mice were generated by Cyagen Biosciences (http://www.cyagen.com/). Their facility meets and often exceeds animal health and welfare guidelines. Animals were euthanized using techniques recommended by the American Veterinary Medical Association. All procedures were carried out in line with Gladstone Institutes and University of California guidelines. All zebrafish work was approved by the UCSF Institutional Animal Care and Use Committee (protocol number AN100466).

### Genomic data

All work presented in this paper is based on the February 2009 assembly of the human genome (GRCh37/hg19) downloaded from the UCSC Genome Browser (http://genome.ucsc.edu/). Any data that was not in reference to this build was mapped over using the *liftOver* tool from the UCSC Kent tools (http://hgdownload.cse.ucsc.edu/admin/jksrc.zip).

### Multiple kernel learning-based prediction of developmental enhancers

In our framework, genomic regions are associated with a common set of descriptive features. We then apply machine-learning algorithms that use the features of known training examples to learn a function of the feature data that distinguishes the positives (enhancers) from the negatives (non-enhancers). This function can then be applied to the features associated with uncharacterized genomic regions to predict their enhancer status. A positive score for a genomic region indicates predicted membership in the positive class (enhancers) and a negative score indicates predicted membership in the negative class (non-enhancers).

#### Training examples

We obtained all of our positive training data and our tissue-specific negative training data from the VISTA Enhancer Browser [69] on April 4, 2012. We downloaded the location, DNA sequence, and expression contexts for all human sequences tested in the VISTA mouse E11.5 enhancer screen. This consisted of 711 validated human enhancers and 736 genomic regions that did not exhibit enhancer activity in this context (http://enhancer.lbl.gov/). The median length of the enhancers in VISTA is 1,545 bp.

In the first step of EnhancerFinder (Figure 1), we used all 711 VISTA enhancers as positive training data. For negative training data, we generated a set of 711 random genomic regions matched to the length and chromosome distribution of the positives, and filtered to remove known VISTA enhancers and assembly gaps.

In the second step of EnhancerFinder, we used tissue-specific subsets of the 1,447 VISTA regions for training. For example, when predicting heart enhancers, our positive training data were the 84 VISTA regions with heart expression in E11.5 mice, and our negative training data were the remaining 1,363 VISTA regions that were tested and showed no heart expression at E11.5, even though they may be enhancers in other tissues or none at all. We did not require that a region be active only in the tissue of interest. We included the VISTA negatives in this analysis, because they share many attributes in common with known enhancers and may have enhancer activity in contexts other than E11.5. Our results did not change dramatically when the VISTA negatives were not included in the training. We trained tissue-specific classifiers for the six tissues with more than 50 examples in VISTA: forebrain, midbrain, hindbrain, heart, limb, and neural tube. We also trained a brain enhancer classifier on the combined the forebrain, midbrain, and hindbrain enhancers.

#### Feature data

We considered three main types of data as features in our analysis: functional genomics data, evolutionary conservation, and DNA sequence motifs. We obtained our functional genomics feature data from the ENCODE data repository at the UCSC Genome Browser (http://genome.ucsc.edu/ENCODE/ and [95]). These data include histone modifications, such as H3K4me1, H3K4me3, H3K27ac, protein-DNA associations for many TFs and p300, and several measurements of open chromatin (DNaseI hypersensitivity, FAIRE, digital genomic footprinting), from hundreds of cell types [95]. We also included heart p300 data from [39]. For a full list of the functional genomics data considered, see Table S1. We associated each genomic region with a binary vector that represents the presence or absence of overlap with each functional genomics data set. To determine this feature vector, we intersected the genomic location of the region of interest with the peaks defined by the original researchers (from the broadPeak or narrowPeak files) using *intersectBed* [96]. We found that considering non-binary functional genomics features based on experimental data, like the density of sequence reads from a ChIP-seq study, did not significantly improve performance (data not shown). However, we suspect that with consistent peak calling and appropriate normalization this might be an avenue for future improvement.

To summarize the DNA sequence motif patterns in a genomic region, we calculated the number of occurrences of all possible 4-mers in the sequence.

Evolutionary conservation estimates were taken from the mammalian phastCons elements [72] obtained from the *phastConsElements46wayPlacental* track in UCSC Genome Browser. Each genomic region was assigned its maximum overlapping phastCons score or zero if it did not overlap any phastCons elements.

#### Machine-learning algorithms

EnhancerFinder is an extension of the SVM supervised learning framework that allows the integration of multiple data types into a single discrimination function. Standard 1-norm MKL augments the usual SVM discrimination function, *f*, with additional parameters, *β_j_*, that weight the contribution of each kernel function *k_j_*:
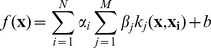
where *N* is the number of training examples, *M* is the number of kernels, *α_i_* are the training example weights, and *b* is the bias [66]. We include three kernel functions in EnhancerFinder, each of which corresponds to one of the three types of feature data described above. These kernels quantify the similarity of the features of the appropriate type for any two genomic regions. To combine the kernels, the MKL algorithm simultaneously learns weights for the associated kernels, in addition to learning the bias and weights for each training example as in a standard SVM. We use the 4-spectrum kernel [71] for our sequence features; this kernel has been shown to perform well in a variety of DNA sequence-based prediction tasks including enhancer prediction [54]. For the functional genomics and evolutionary conservation data, we use linear kernels, which are equivalent to dot products of the feature vectors. We explored the use of alternative, non-linear kernels for these features and found that they performed similarly (data not shown). Each kernel was variance normalized, and we balanced the misclassification costs by class size [97]. In addition to EnhancerFinder classifiers, we also trained and evaluated the constituent single kernel SVMs. All analyses were performed using the implementation of SVMs and MKL in the SHOGUN Machine Learning Toolbox v1.1.0 [98].

### Performance evaluations

To evaluate the performance of trained classifiers, we performed 10-fold cross-validation on the training data and quantified our results with ROC AUC, precision-recall curves, and power estimates at fixed false positive rates. We computed p-values for the difference in performance between classification methods using McNemar's test [99], [100]. To estimate false discovery rates, we trained EnhancerFinder classifiers at 1∶1, 1∶10, and 1∶100 ratios of positive to negative enhancers and used the resulting 10-fold cross-validation results to calculate the proportion of false discoveries genome-wide at a 5% FPR if the true proportion of 1.5 kb windows containing an enhancer was 50%, 10%, or 1%.

### Comparison to existing enhancer prediction methods

We compared EnhancerFinder's predictions to those of several previous enhancer prediction methods. We obtained the performance of CLARE on our Step 1 prediction task, by inputting our positive and negative data into the CLARE web server [73]. We downloaded the genomic segmentations and annotations produced by ChromHMM [64] and Segway [65]. We considered the ChromHMM predictions based on different ENCODE cell lines both individually and together. Any genomic region in our evaluation data set that overlapped an enhancer state was considered a predicted enhancer, and all others were considered predicted non-enhancers. For Segway, we also considered the “TF activity” state.

### Identification of tissue-specific enhancers across the human genome

We predicted tissue-specific developmental enhancers throughout the human genome by applying a trained MKL classifier (Step 1 of EnhancerFinder) without conservation (see Results) to sliding windows of 1500 bp, moving along the human genome in 500 bp steps. The feature profile for each window was computed as described above. To focus on high-confidence predictions, we filtered the enhancer scores for the windows at a 5% FPR, estimated from cross-validation using the genomic background, and combined the remaining overlapping windows to produce 84,301 high-confidence predicted enhancers.

To predict tissue specificity, we applied trained brain, limb, and heart classifiers (Step 2 of EnhancerFinder) without conservation to all 299,039 windows with positive enhancer scores in Step 1. We then applied a 5% FPR cutoff for each tissue and concatenated the remaining overlapping windows into merged enhancer regions. Using this approach, we predicted 19,051 heart enhancers, 11,693 brain enhancers, and 7,400 limb enhancers.

### Analysis of genome-wide tissue-specific enhancer predictions

We characterized the expression patterns of the gene nearest to each predicted enhancer using the GNF Atlas 2 [101]. It contains expression data for genes in 79 different tissues, with expression measured using Affymetrix microarrays. For each of these 79 tissues, we used a paired t-test to determine if the nearest genes of predicted heart enhancers had significantly different mean values of expression than the nearest genes of brain enhancers. We did not include the limb enhancers in this analysis due to the lack of relevant expression data in the GNF Atlas 2.

We examined genomic regions near predicted developmental enhancers for enrichment of Gene Ontology functional annotations, known phenotypes, and pathways using GREAT [102]. Results were computed using the hypergeometric test for genome-wide significance, with the default settings and the “basal plus extension” association rule (proximal 5 kb upstream, 1 kb downstream, plus distal up to 100 kb).

We identified the sequence motifs present in each set of enhancers using the FIMO tool (Find Individual Motif Occurrences) from the MEME Suite of sequence motif analysis tools [103]. We considered known transcription factor binding motifs from the April 2011 release of the TRANSFAC database with a FIMO score threshold of 10e-5. We identified those occurrences that fell in predicted enhancers, and summarized motifs to identify the most prevalent TFs in each tissue-specific set of enhancers.

We analyzed the overlap of predicted enhancers with GWAS SNPs, based on the NHGRI catalog of 9,687 GWAS SNPs downloaded from the UCSC Genome Browser in October 2012. Unadjusted permutation p-values were calculated by randomizing genomic locations of predicted enhancers (matching for length and chromosome, and avoiding assembly gaps) and overlapping these randomized regions with GWAS SNPs to assess significance of overlapping regions.

### Transgenic enhancer assays

Mouse enhancer assays were carried out in transient transgenic mouse embryos generated by pronuclear injections of enhancer assay constructs into FVB embryos (Cyagen Biosciences). Human and chimpanzee DNA sequences were inserted upstream of a minimal promoter Hsp68 and a *LacZ* reporter gene. The human sequence was amplified using primers 5′-TGTATGAAACCTGTTCACTCTCC-3′ and 5′-GCTTAAAACAACTACTAGAATCAGGC-3′ from the bacterial artificial chromosome (BAC) RP11-107E5 (from the BacPac resource at CHORI). The chimpanzee sequence was amplified using primers 5′-TGTATGAAACCTGTTCACTCTCC-3′ and 5′-GCTTAAAACAACTACTAGAATCAGGC-3′ from BAC CH251-677E03a (CHORI). The embryos were collected and stained for *LacZ* expression at E11.5.

Following the annotation policies of the VISTA Enhancer Browser, we required that consistent spatial expression patterns be present in three or more embryos with staining in order for the region to be considered an enhancer.

Zebrafish enhancer assays were performed in transient transgenic zebrafish embryos. We tested candidate enhancer regions that ranged in length from 987 bp to 3,633 bp (see Table S6 for hg19 genomic coordinates), which we manually demarcated from within larger predicted enhancer regions based on signatures of likely enhancer function (including DnaseI hypersensitivity sites, transcription factor binding sites, histone modifications, and conservation).

We performed PCR to obtain the candidate enhancer sequence using human genomic DNA (Roche). These were cloned into the E1b-GFP-Tol2 enhancer assay vector containing an E1b minimal promoter followed by GFP [104], and the construct was verified by sequencing. Each construct was injected with Tol2 mRNA into at least 100 single-cell fertilized zebrafish embryos. We annotated GFP expression at approximately 24 and 48 hours post fertilization (hpf), and considered an enhancer to be positive if we observed consistent expression in at least 15% of all fish alive at either 24 or 48 hpf [105], and suggestive of enhancer activity if we observed consistent expression in at least 10% of all fish alive at 24 or 48 hpf, after subtracting out percentages of tissue expression in fish injected with the empty enhancer vector. For each construct, at least 50 fish were analyzed for GFP expression at 48 hpf.

## Supporting Information

Figure S1 **Precision-Recall curves corresponding to all ROC curves presented in the main text.** (A) Figure 2A (B) Figure 3A (C) Figure 3B (D) Figure 4. A PR curve could not be created for Figure 2C, because we could not obtain the raw scores for regions from the CLARE web server.
(PDF)

Figure S2 **VISTA enhancers overlap many common marks of enhancers, but no common mark is universal to all VISTA enhancers.** We computed the overlap between 711 VISTA enhancers and three common functional genomic marks of enhancers and found that 450 enhancers overlap H3K27ac (in any of 16 datasets from ENCODE), 563 overlap H3K4me1 (in any of 15 datasets from ENCODE), and 404 overlap p300/CBP (in any of 35 datasets from ENCODE and human tissues). Fewer than half of the enhancers (306) overlap all three common marks of enhancers, and 93 do not overlap any of those three functional genomics marks. All but five of the VISTA enhancers overlap a conservation peak (phastCons 46-way placental mammal). Four of these non-conserved enhancers overlap all three functional genomics marks, and one non-conserved enhancer overlaps just H3K27ac and H3K4me1.
(PDF)

Figure S3 **The 4-spectrum kernel performs competitively with other k-spectrum kernels and the combination of k-spectrum kernels.** We analyzed the ability of spectrum kernels based on k-mer lengths between 2 and 8 to distinguish enhancers from the genomic background (Step 1). K-mers between 4 and 7 had the best performance. We also evaluated an MKL algorithm that combined each k-spectrum kernel, and it did not provide significant improvement over the best individual kernels.
(PDF)

Figure S4 **Considering known TFBS motifs does not improve the 4-spectrum kernel.** Considering the number of occurrences of known TFBS motifs as features has recently been used in a linear SVM framework to predict enhancers [52]. To evaluate the utility of this approach, instead of and in addition to considering all k-mers, we created a linear SVM that used the number of hits to 1022 TF binding site matrices from TRANSFAC and JASPAR as computed by FIMO as features. That is the feature vector for each region consisted of 1022 elements, each of which was the number of significant hits for a different TF motif. This TFBS linear SVM (AUC = 0.81) did not perform as well as the 4-spectrum kernel (AUC = 0.88). We also evaluated an MKL algorithm that combined the 4-spectrum and TFBS kernels. This combined kernel did not perform any better than the 4-spectrum kernel suggesting that, at least under this encoding, TFBS motifs do not provide significant additional benefit in distinguishing enhancers from the genomic background.
(PDF)

Figure S5 **Combining functional genomics data with an SVM outperforms simply considering regions overlapping these data.** The four solid lines shown are the same as in Figure 3B; they summarize the performance of these methods at distinguishing VISTA enhancers from the genomic background (Step 1). The X's give the performance of approaches that consider all regions overlapping a given feature as positives and all others as negatives. The + and * indicate the performance obtained by considering the union and intersection of H3K4me1, p300, and H3K27ac, respectively. For each feature, the linear SVM achieves better performance than simply considering all overlapping regions as positives.
(PDF)

Figure S6 **EnhancerFinder feature weights highlight the contribution of different functional genomics data types to enhancer predictions.** Each “+” represents the contribution made by a single data feature, e.g. H3K4me1 peaks from embryonic stem cells, to the classification in EnhancerFinder Step 1 (developmental enhancers versus genomic background). Positive weights (red) indicate an association with enhancer activity in our analysis and negative weights (blue) suggest a lack of enhancer activity. The features plotted here come from a range of likely relevant contexts (**Relevant Functional Genomics** classifier; Table S1), and the number of data sets present for each feature type is given in parentheses. The black bar gives the average weight over all features of each type. In general, the features with high average weights, such as H3K3me1, p300, and H3K4me2, are known to be associated with enhancers, while those with large negative weights are associated with other types of genomic regions. However, no data type has uniformly positive or negative weights in all contexts.
(PDF)

Figure S7 **Heart enhancers are less conserved and closer to the nearest transcription start site (TSS) than limb and brain enhancers.** Considering only limb and brain enhancers that are less evolutionarily conserved and close to a TSS improved our ability to identify them, but they are still more difficult to identify than heart enhancers. In addition to these features, heart enhancers have uniquely high GC content compared to other enhancers and the genomic background (Figure S7).
(PDF)

Figure S8 **The uniquely high GC content of heart enhancers in VISTA enables accurate classification.** The VISTA heart enhancers have higher GC content (49%) than other types of enhancers and the genomic background (∼40%). (A) The classification score from a spectrum kernel classifier trained to distinguish heart enhancers within VISTA (Step 2) is strongly correlated (Pearson rho = 0.95) with the GC content of the input region. (B) A classification algorithm based solely on GC content (black) performs competitively with the spectrum kernel (AUC of 0.80 vs. 0.82), and nearly as well as EnhancerFinder (0.85; Figure 4).
(PDF)

Figure S9 **Enhancers active in multiple tissues are easier to identify than those active in a single tissue.** There are 399 enhancers active in a single tissue at E11.5 in the VISTA database and 312 active in multiple tissues. EnhancerFinder is better able to distinguish the enhancers active in multiple tissues from the VISTA negatives (AUC = 0.75) than it is to distinguish single tissue enhancers from the negatives (AUC = 0.67). This trend also holds across each tissue individually. However, both sets are easy to distinguish from the genomic background (AUC = 0.96 for both, not shown).
(PDF)

Figure S10 **Three novel developmental enhancers near** ***FOXC1*** **.** This UCSC Genome Browser screenshot shows six candidate enhancer regions tested in transgenic zebrafish. Three of the regions showed positive or suggestive expression at 24 or 48 hpf. F1EC-1 drives expression at 48 hpf; the arrows highlight reproducible midbrain, spinal cord, and epidermis expression. F1EC-3 shows suggestive expression at 24 hpf in somitic muscles and the epidermis (arrows). F1EC-6 drives expression at 48 hpf in the pericardium and heart (suggestive). The other three tested candidate enhancers without corresponding zebrafish images were negative in the enhancer assay. See Table S6 for full list of expressed tissues seen in each candidate enhancer.
(PDF)

Figure S11 **Transient transgenic mouse embryos support a novel cranial nerve enhancer near** ***ZEB2*** **.** Seven transient transgenic mouse embryos showed *LacZ* expression at embryonic day 11.5. Constructs containing a 999 bp region (hg19.chr2:145,234,541–145,235,539) including 2xHAR.240 near *ZEB2*, a minimal promoter, and *LacZ* were used for human. The orthologous region was used in the chimp construct (panTro2.chr2b:148,811,929–148,812,929). Three embryos with constructs containing the human version of the region of interest and four embryos containing the chimp sequence had staining. In all embryos, there was consistent expression in the cranial nerve. There does not appear to be a significant difference in the activity driven by the human and chimp sequences at this time point.
(PDF)

Table S1 **Functional genomics features used in our analysis.** This Excel spreadsheet lists the files used from ENCODE (http://genome.ucsc.edu/ENCODE/) or GEO (http://www.ncbi.nlm.nih.gov/geo/). There is a sheet for each of the classifiers based on functional genomics data that lists all data files used. ENCODE data set names are UCSC track names. GEO data set names are GEO identifiers.
(XLS)

Table S2 **Genes near brain enhancers have significantly higher gene expression in brain and neural tissues than genes near heart enhancers.** Brain- or heart-related tissues with significantly higher mean expression in genes associated with predicted brain enhancers compared to predicted heart enhancers.
(DOC)

Table S3 **Genes near heart enhancers have significantly higher gene expression in cardiac-related tissues than genes near brain enhancers.** Brain- or heart-related tissues with significantly higher mean expression in genes associated with predicted heart enhancers compared to predicted brain enhancers.
(DOC)

Table S4 **The top 25 transcription factors for which binding sites were most prevalent in brain, heart, and limb enhancers.**
(DOC)

Table S5 **676 GWAS SNPs are found in predicted enhancers.** This Excel spreadsheet lists all GWAS SNPs from the NHGRI database that fall within one of our predicted enhancers.
(XLSX)

Table S6 **Candidate enhancer regions tested in zebrafish.** We tested 10 candidate enhancer regions in a transgenic zebrafish assay. This table lists the genomic coordinates (hg19) and expression patterns observed for each construct at 24 and 48 hpf. A representative fish is shown for each positive enhancer in (Figures 7 and S9). Candidate enhancers on chromosome 6 are near FOXC1, and those on chromosome 16 are near FOXC2. N is the number of zebrafish alive at the specified time point, and * indicates expression patterns that are “suggestive,” but below the 15% threshold we used for confirmed enhancers.
(DOC)

Data File S1 **This ZIP archive contains BED files (hg19 coordinates) with EnhancerFinder's genome-wide enhancer predictions, along with the MKL scores, for general developmental enhancer activity, brain, heart, and limb enhancers.** The general prediction file also lists the H3K27ac and H3K4me1 marks from the feature data overlapping each predicted enhancer.
(ZIP)

Text S1 **Text describing additional analyses in support of the manuscript.**
(DOC)

## References

[1] Ong CT, Corces VG (2011) Enhancer function: new insights into the regulation of tissue-specific gene expression. Nature reviews Genetics 12: 283–293. 10.1038/nrg2957 PMC3175006 21358745

[2] Bulger M, Groudine M (2011) Functional and mechanistic diversity of distal transcription enhancers. Cell 144: 327–339. 21295696 10.1016/j.cell.2011.01.024 PMC3742076

[3] Visel A, Rubin EM, Pennacchio LA (2009) Genomic views of distant-acting enhancers. Nature 461: 199–205. 19741700 10.1038/nature08451 PMC2923221

[4] Sakabe NJ, Savic D, Nobrega MA (2012) Transcriptional enhancers in development and disease. Genome biology 13: 238. 22269347 10.1186/gb-2012-13-1-238 PMC3334578

[5] Ahituv N (2012) Gene regulatory sequences and human disease. New York: Springer. x, 283 pages p.

[6] Noonan JP, McCallion AS (2010) Genomics of long-range regulatory elements. Annual review of genomics and human genetics 11: 1–23. 10.1146/annurev-genom-082509-141651 20438361

[7] Lomvardas S, Barnea G, Pisapia DJ, Mendelsohn M, Kirkland J, et al (2006) Interchromosomal interactions and olfactory receptor choice. Cell 126: 403–413. 16873069 10.1016/j.cell.2006.06.035

[8] Visel A, Akiyama JA, Shoukry M, Afzal V, Rubin EM, et al (2009) Functional autonomy of distant-acting human enhancers. Genomics 93: 509–513. 19268701 10.1016/j.ygeno.2009.02.002 PMC2683195

[9] Visel A, Taher L, Girgis H, May D, Golonzhka O, et al (2013) A high-resolution enhancer atlas of the developing telencephalon. Cell 152: 895–908. 23375746 10.1016/j.cell.2012.12.041 PMC3660042

[10] Koch CM, Andrews RM, Flicek P, Dillon SC, Karaoz U, et al (2007) The landscape of histone modifications across 1% of the human genome in five human cell lines. Genome research 17: 691–707. 17567990 10.1101/gr.5704207 PMC1891331

[11] Heintzman ND, Hon GC, Hawkins RD, Kheradpour P, Stark A, et al (2009) Histone modifications at human enhancers reflect global cell-type-specific gene expression. Nature 459: 108–112. 19295514 10.1038/nature07829 PMC2910248

[12] Sholtis SJ, Noonan JP (2010) Gene regulation and the origins of human biological uniqueness. Trends in genetics : TIG 26: 110–118. 20106546 10.1016/j.tig.2009.12.009

[13] Levine M (2010) Transcriptional enhancers in animal development and evolution. Current biology : CB 20: R754–763. 20833320 10.1016/j.cub.2010.06.070 PMC4280268

[14] Banerji J, Rusconi S, Schaffner W (1981) Expression of a beta-globin gene is enhanced by remote SV40 DNA sequences. Cell 27: 299–308. 6277502 10.1016/0092-8674(81)90413-x

[15] Gillies SD, Morrison SL, Oi VT, Tonegawa S (1983) A tissue-specific transcription enhancer element is located in the major intron of a rearranged immunoglobulin heavy chain gene. Cell 33: 717–728. 6409417 10.1016/0092-8674(83)90014-4

[16] Nobrega MA, Ovcharenko I, Afzal V, Rubin EM (2003) Scanning human gene deserts for long-range enhancers. Science 302: 413. 14563999 10.1126/science.1088328

[17] Pennacchio LA, Ahituv N, Moses AM, Prabhakar S, Nobrega MA, et al (2006) In vivo enhancer analysis of human conserved non-coding sequences. Nature 444: 499–502. 17086198 10.1038/nature05295

[18] Visel A, Blow MJ, Li Z, Zhang T, Akiyama JA, et al (2009) ChIP-seq accurately predicts tissue-specific activity of enhancers. Nature 457: 854–858. 19212405 10.1038/nature07730 PMC2745234

[19] Visel A, Prabhakar S, Akiyama JA, Shoukry M, Lewis KD, et al (2008) Ultraconservation identifies a small subset of extremely constrained developmental enhancers. Nature genetics 40: 158–160. 18176564 10.1038/ng.2007.55 PMC2647775

[20] Woolfe A, Goodson M, Goode DK, Snell P, McEwen GK, et al (2005) Highly conserved non-coding sequences are associated with vertebrate development. PLoS biology 3: e7. 15630479 10.1371/journal.pbio.0030007 PMC526512

[21] Prabhakar S, Poulin F, Shoukry M, Afzal V, Rubin EM, et al (2006) Close sequence comparisons are sufficient to identify human cis-regulatory elements. Genome research 16: 855–863. 16769978 10.1101/gr.4717506 PMC1484452

[22] McGaughey DM, Vinton RM, Huynh J, Al-Saif A, Beer MA, et al (2008) Metrics of sequence constraint overlook regulatory sequences in an exhaustive analysis at phox2b. Genome research 18: 252–260. 18071029 10.1101/gr.6929408 PMC2203623

[23] Johnson DS, Mortazavi A, Myers RM, Wold B (2007) Genome-wide mapping of in vivo protein-DNA interactions. Science 316: 1497–1502. 17540862 10.1126/science.1141319

[24] Boyle AP, Davis S, Shulha HP, Meltzer P, Margulies EH, et al (2008) High-resolution mapping and characterization of open chromatin across the genome. Cell 132: 311–322. 18243105 10.1016/j.cell.2007.12.014 PMC2669738

[25] Giresi PG, Kim J, McDaniell RM, Iyer VR, Lieb JD (2007) FAIRE (Formaldehyde-Assisted Isolation of Regulatory Elements) isolates active regulatory elements from human chromatin. Genome research 17: 877–885. 17179217 10.1101/gr.5533506 PMC1891346

[26] Dunham I, Kundaje A, Aldred SF, Collins PJ, Davis CA, et al (2012) An integrated encyclopedia of DNA elements in the human genome. Nature 489: 57–74. 22955616 10.1038/nature11247 PMC3439153

[27] Andersson R, Gebhard C, Miguel-Escalada I, Hoof I, Bornholdt J, et al (2014) An atlas of active enhancers across human cell types and tissues. Nature 507: 455–461. 24670763 10.1038/nature12787 PMC5215096

[28] Wamstad JA, Alexander JM, Truty RM, Shrikumar A, Li F, et al (2012) Dynamic and coordinated epigenetic regulation of developmental transitions in the cardiac lineage. Cell 151: 206–220. 22981692 10.1016/j.cell.2012.07.035 PMC3462286

[29] Paige SL, Thomas S, Stoick-Cooper CL, Wang H, Maves L, et al (2012) A temporal chromatin signature in human embryonic stem cells identifies regulators of cardiac development. Cell 151: 221–232. 22981225 10.1016/j.cell.2012.08.027 PMC3462257

[30] Jin C, Zang C, Wei G, Cui K, Peng W, et al (2009) H3.3/H2A.Z double variant-containing nucleosomes mark ‘nucleosome-free regions’ of active promoters and other regulatory regions. Nature genetics 41: 941–945. 19633671 10.1038/ng.409 PMC3125718

[31] He HH, Meyer CA, Shin H, Bailey ST, Wei G, et al (2010) Nucleosome dynamics define transcriptional enhancers. Nature genetics 42: 343–347. 20208536 10.1038/ng.545 PMC2932437

[32] Thurman RE, Rynes E, Humbert R, Vierstra J, Maurano MT, et al (2012) The accessible chromatin landscape of the human genome. Nature 489: 75–82. 22955617 10.1038/nature11232 PMC3721348

[33] Heintzman ND, Stuart RK, Hon G, Fu Y, Ching CW, et al (2007) Distinct and predictive chromatin signatures of transcriptional promoters and enhancers in the human genome. Nature genetics 39: 311–318. 17277777 10.1038/ng1966

[34] Cotney J, Leng J, Oh S, DeMare LE, Reilly SK, et al (2012) Chromatin state signatures associated with tissue-specific gene expression and enhancer activity in the embryonic limb. Genome research 22: 1069–1080. 22421546 10.1101/gr.129817.111 PMC3371702

[35] Creyghton MP, Cheng AW, Welstead GG, Kooistra T, Carey BW, et al (2010) Histone H3K27ac separates active from poised enhancers and predicts developmental state. Proceedings of the National Academy of Sciences of the United States of America 107: 21931–21936. 21106759 10.1073/pnas.1016071107 PMC3003124

[36] Rada-Iglesias A, Bajpai R, Swigut T, Brugmann SA, Flynn RA, et al (2011) A unique chromatin signature uncovers early developmental enhancers in humans. Nature 470: 279–283. 21160473 10.1038/nature09692 PMC4445674

[37] Mikkelsen TS, Ku M, Jaffe DB, Issac B, Lieberman E, et al (2007) Genome-wide maps of chromatin state in pluripotent and lineage-committed cells. Nature 448: 553–560. 17603471 10.1038/nature06008 PMC2921165

[38] Zhou VW, Goren A, Bernstein BE (2011) Charting histone modifications and the functional organization of mammalian genomes. Nature reviews Genetics 12: 7–18. 10.1038/nrg2905 21116306

[39] Blow MJ, McCulley DJ, Li Z, Zhang T, Akiyama JA, et al (2010) ChIP-Seq identification of weakly conserved heart enhancers. Nature genetics 42: 806–810. 20729851 10.1038/ng.650 PMC3138496

[40] Ghisletti S, Barozzi I, Mietton F, Polletti S, De Santa F, et al (2010) Identification and characterization of enhancers controlling the inflammatory gene expression program in macrophages. Immunity 32: 317–328. 20206554 10.1016/j.immuni.2010.02.008

[41] May D, Blow MJ, Kaplan T, McCulley DJ, Jensen BC, et al (2012) Large-scale discovery of enhancers from human heart tissue. Nature genetics 44: 89–93. 22138689 10.1038/ng.1006 PMC3246570

[42] Zinzen RP, Girardot C, Gagneur J, Braun M, Furlong EE (2009) Combinatorial binding predicts spatio-temporal cis-regulatory activity. Nature 462: 65–70. 19890324 10.1038/nature08531

[43] He A, Kong SW, Ma Q, Pu WT (2011) Co-occupancy by multiple cardiac transcription factors identifies transcriptional enhancers active in heart. Proceedings of the National Academy of Sciences of the United States of America 108: 5632–5637. 21415370 10.1073/pnas.1016959108 PMC3078411

[44] Yip KY, Cheng C, Bhardwaj N, Brown JB, Leng J, et al (2012) Classification of human genomic regions based on experimentally determined binding sites of more than 100 transcription-related factors. Genome biology 13: R48. 22950945 10.1186/gb-2012-13-9-r48 PMC3491392

[45] Cheng C, Alexander R, Min R, Leng J, Yip KY, et al (2012) Understanding transcriptional regulation by integrative analysis of transcription factor binding data. Genome research 22: 1658–1667. 22955978 10.1101/gr.136838.111 PMC3431483

[46] Orom UA, Derrien T, Beringer M, Gumireddy K, Gardini A, et al (2010) Long noncoding RNAs with enhancer-like function in human cells. Cell 143: 46–58. 20887892 10.1016/j.cell.2010.09.001 PMC4108080

[47] Barski A, Cuddapah S, Cui K, Roh TY, Schones DE, et al (2007) High-resolution profiling of histone methylations in the human genome. Cell 129: 823–837. 17512414 10.1016/j.cell.2007.05.009

[48] Wang Z, Zang C, Rosenfeld JA, Schones DE, Barski A, et al (2008) Combinatorial patterns of histone acetylations and methylations in the human genome. Nature genetics 40: 897–903. 18552846 10.1038/ng.154 PMC2769248

[49] Zentner GE, Tesar PJ, Scacheri PC (2011) Epigenetic signatures distinguish multiple classes of enhancers with distinct cellular functions. Genome research 21: 1273–1283. 21632746 10.1101/gr.122382.111 PMC3149494

[50] Bonn S, Zinzen RP, Girardot C, Gustafson EH, Perez-Gonzalez A, et al (2012) Tissue-specific analysis of chromatin state identifies temporal signatures of enhancer activity during embryonic development. Nature genetics 44: 148–156. 22231485 10.1038/ng.1064

[51] Narlikar L, Sakabe NJ, Blanski AA, Arimura FE, Westlund JM, et al (2010) Genome-wide discovery of human heart enhancers. Genome research 20: 381–392. 20075146 10.1101/gr.098657.109 PMC2840982

[52] Burzynski GM, Reed X, Taher L, Stine ZE, Matsui T, et al (2012) Systematic elucidation and in vivo validation of sequences enriched in hindbrain transcriptional control. Genome research 22: 2278–2289. 22759862 10.1101/gr.139717.112 PMC3483557

[53] Busser BW, Taher L, Kim Y, Tansey T, Bloom MJ, et al (2012) A machine learning approach for identifying novel cell type-specific transcriptional regulators of myogenesis. PLoS genetics 8: e1002531. 22412381 10.1371/journal.pgen.1002531 PMC3297574

[54] Lee D, Karchin R, Beer MA (2011) Discriminative prediction of mammalian enhancers from DNA sequence. Genome research 21: 2167–2180. 21875935 10.1101/gr.121905.111 PMC3227105

[55] Gorkin DU, Lee D, Reed X, Fletez-Brant C, Bessling SL, et al (2012) Integration of ChIP-seq and machine learning reveals enhancers and a predictive regulatory sequence vocabulary in melanocytes. Genome research 22: 2290–2301. 23019145 10.1101/gr.139360.112 PMC3483558

[56] Rajagopal N, Xie W, Li Y, Wagner U, Wang W, et al (2013) RFECS: a random-forest based algorithm for enhancer identification from chromatin state. PLoS computational biology 9: e1002968. 23526891 10.1371/journal.pcbi.1002968 PMC3597546

[57] Lahdesmaki H, Rust AG, Shmulevich I (2008) Probabilistic inference of transcription factor binding from multiple data sources. PloS one 3: e1820. 18364997 10.1371/journal.pone.0001820 PMC2268002

[58] Kantorovitz MR, Kazemian M, Kinston S, Miranda-Saavedra D, Zhu Q, et al (2009) Motif-blind, genome-wide discovery of cis-regulatory modules in Drosophila and mouse. Developmental cell 17: 568–579. 19853570 10.1016/j.devcel.2009.09.002 PMC2768654

[59] Won KJ, Ren B, Wang W (2010) Genome-wide prediction of transcription factor binding sites using an integrated model. Genome biology 11: R7. 20096096 10.1186/gb-2010-11-1-r7 PMC2847719

[60] Pique-Regi R, Degner JF, Pai AA, Gaffney DJ, Gilad Y, et al (2011) Accurate inference of transcription factor binding from DNA sequence and chromatin accessibility data. Genome research 21: 447–455. 21106904 10.1101/gr.112623.110 PMC3044858

[61] Arvey A, Agius P, Noble WS, Leslie C (2012) Sequence and chromatin determinants of cell-type-specific transcription factor binding. Genome research 22: 1723–1734. 22955984 10.1101/gr.127712.111 PMC3431489

[62] Cuellar-Partida G, Buske FA, McLeay RC, Whitington T, Noble WS, et al (2012) Epigenetic priors for identifying active transcription factor binding sites. Bioinformatics 28: 56–62. 22072382 10.1093/bioinformatics/btr614 PMC3244768

[63] Wang D, Do HT (2012) Computational localization of transcription factor binding sites using extreme learning machines. Soft Comput 16: 1595–1606.

[64] Ernst J, Kheradpour P, Mikkelsen TS, Shoresh N, Ward LD, et al (2011) Mapping and analysis of chromatin state dynamics in nine human cell types. Nature 473: 43–49. 21441907 10.1038/nature09906 PMC3088773

[65] Hoffman MM, Buske OJ, Wang J, Weng Z, Bilmes JA, et al (2012) Unsupervised pattern discovery in human chromatin structure through genomic segmentation. Nature methods 9: 473–476. 22426492 10.1038/nmeth.1937 PMC3340533

[66] Sonnenburg S, Zien A, Ratsch G (2006) ARTS: accurate recognition of transcription starts in human. Bioinformatics 22: e472–480. 16873509 10.1093/bioinformatics/btl250

[67] Kloft M, Brefeld U, Sonnenburg S, Zien A (2011) lp-Norm Multiple Kernel Learning. Journal of Machine Learning Research 12: 953–997.

[68] Boser BE, Guyon IM, Vapnik VN (1992) A training algorithm for optimal margin classifiers. Proceedings of the fifth annual workshop on Computational learning theory. Pittsburgh, Pennsylvania, USA: ACM. pp. 144–152.

[69] Visel A, Minovitsky S, Dubchak I, Pennacchio LA (2007) VISTA Enhancer Browser–a database of tissue-specific human enhancers. Nucleic acids research 35: D88–92. 17130149 10.1093/nar/gkl822 PMC1716724

[70] O'Rahilly R, Muller F (2010) Developmental stages in human embryos: revised and new measurements. Cells, tissues, organs 192: 73–84. 20185898 10.1159/000289817

[71] Leslie C, Eskin E, Noble WS (2002) The spectrum kernel: a string kernel for SVM protein classification. Pacific Symposium on Biocomputing Pacific Symposium on Biocomputing 564–575. 11928508

[72] Siepel A, Bejerano G, Pedersen JS, Hinrichs AS, Hou M, et al (2005) Evolutionarily conserved elements in vertebrate, insect, worm, and yeast genomes. Genome research 15: 1034–1050. 16024819 10.1101/gr.3715005 PMC1182216

[73] Taher L, Narlikar L, Ovcharenko I (2012) CLARE: Cracking the LAnguage of Regulatory Elements. Bioinformatics 28: 581–583. 22199387 10.1093/bioinformatics/btr704 PMC3278760

[74] Capra JA, Erwin GD, McKinsey G, Rubenstein JLR, Pollard KS (2013) Many human accelerated regions are developmental enhancers. Philos Trans R Soc Lond B Biol Sci 10.1098/rstb.2013.0025 PMC3826498 24218637

[75] Nord AS, Blow MJ, Attanasio C, Akiyama JA, Holt A, et al (2013) Rapid and Pervasive Changes in Genome-wide Enhancer Usage during Mammalian Development. Cell 155: 1521–1531. 24360275 10.1016/j.cell.2013.11.033 PMC3989111

[76] Hindorff LA, Sethupathy P, Junkins HA, Ramos EM, Mehta JP, et al (2009) Potential etiologic and functional implications of genome-wide association loci for human diseases and traits. Proceedings of the National Academy of Sciences of the United States of America 106: 9362–9367. 19474294 10.1073/pnas.0903103106 PMC2687147

[77] Kume T, Deng K, Hogan BL (2000) Murine forkhead/winged helix genes Foxc1 (Mf1) and Foxc2 (Mfh1) are required for the early organogenesis of the kidney and urinary tract. Development 127: 1387–1395. 10704385 10.1242/dev.127.7.1387

[78] Kume T, Jiang H, Topczewska JM, Hogan BL (2001) The murine winged helix transcription factors, Foxc1 and Foxc2, are both required for cardiovascular development and somitogenesis. Genes & development 15: 2470–2482. 11562355 10.1101/gad.907301 PMC312788

[79] Maiese K (2010) Forkhead Transcription Factors. New York: Springer.

[80] Smith RS, Zabaleta A, Kume T, Savinova OV, Kidson SH, et al (2000) Haploinsufficiency of the transcription factors FOXC1 and FOXC2 results in aberrant ocular development. Human molecular genetics 9: 1021–1032. 10767326 10.1093/hmg/9.7.1021

[81] Aldinger KA, Lehmann OJ, Hudgins L, Chizhikov VV, Bassuk AG, et al (2009) FOXC1 is required for normal cerebellar development and is a major contributor to chromosome 6p25.3 Dandy-Walker malformation. Nature genetics 41: 1037–1042. 19668217 10.1038/ng.422 PMC2843139

[82] Seuntjens E, Nityanandam A, Miquelajauregui A, Debruyn J, Stryjewska A, et al (2009) Sip1 regulates sequential fate decisions by feedback signaling from postmitotic neurons to progenitors. Nature neuroscience 12: 1373–1380. 19838179 10.1038/nn.2409

[83] Miquelajauregui A, Van de Putte T, Polyakov A, Nityanandam A, Boppana S, et al (2007) Smad-interacting protein-1 (Zfhx1b) acts upstream of Wnt signaling in the mouse hippocampus and controls its formation. Proceedings of the National Academy of Sciences of the United States of America 104: 12919–12924. 17644613 10.1073/pnas.0609863104 PMC1929013

[84] Weng Q, Chen Y, Wang H, Xu X, Yang B, et al (2012) Dual-mode modulation of Smad signaling by Smad-interacting protein Sip1 is required for myelination in the central nervous system. Neuron 73: 713–728. 22365546 10.1016/j.neuron.2011.12.021 PMC3293152

[85] Renthal NE, Chen CC, Williams KC, Gerard RD, Prange-Kiel J, et al (2010) miR-200 family and targets, ZEB1 and ZEB2, modulate uterine quiescence and contractility during pregnancy and labor. Proceedings of the National Academy of Sciences of the United States of America 107: 20828–20833. 21079000 10.1073/pnas.1008301107 PMC2996411

[86] Wilson M, Mowat D, Dastot-Le Moal F, Cacheux V, Kaariainen H, et al (2003) Further delineation of the phenotype associated with heterozygous mutations in ZFHX1B. American journal of medical genetics Part A 119A: 257–265. 12784289 10.1002/ajmg.a.20053

[87] El-Kasti MM, Wells T, Carter DA (2012) A novel long-range enhancer regulates postnatal expression of Zeb2: implications for Mowat-Wilson syndrome phenotypes. Human molecular genetics 21: 5429–5442. 23001561 10.1093/hmg/dds389

[88] Pollard KS, Salama SR, King B, Kern AD, Dreszer T, et al (2006) Forces shaping the fastest evolving regions in the human genome. PLoS genetics 2: e168. 17040131 10.1371/journal.pgen.0020168 PMC1599772

[89] Lindblad-Toh K, Garber M, Zuk O, Lin MF, Parker BJ, et al (2011) A high-resolution map of human evolutionary constraint using 29 mammals. Nature 478: 476–482. 21993624 10.1038/nature10530 PMC3207357

[90] Capra JA, Erwin GD, McKinsey G, Rubenstein JLR, Pollard KS (2013) Many human accelerated regions are developmental enhancers. Philosophical Transactions of the Royal Society B: Biological Sciences 3681: 1632. 10.1098/rstb.2013.0025 PMC3826498 24218637

[91] Woznica A, Haeussler M, Starobinska E, Jemmett J, Li Y, et al (2012) Initial deployment of the cardiogenic gene regulatory network in the basal chordate, Ciona intestinalis. Developmental biology 368: 127–139. 22595514 10.1016/j.ydbio.2012.05.002 PMC3383925

[92] Koshiba-Takeuchi K, Mori AD, Kaynak BL, Cebra-Thomas J, Sukonnik T, et al (2009) Reptilian heart development and the molecular basis of cardiac chamber evolution. Nature 461: 95–98. 19727199 10.1038/nature08324 PMC2753965

[93] Casci T (2011) Development: Hourglass theory gets molecular approval. Nature reviews Genetics 12: 76. 10.1038/nrg2940 21173773

[94] White MA, Myers CA, Corbo JC, Cohen BA (2013) Massively parallel in vivo enhancer assay reveals that highly local features determine the cis-regulatory function of ChIP-seq peaks. Proceedings of the National Academy of Sciences of the United States of America 110: 11952–11957. 23818646 10.1073/pnas.1307449110 PMC3718143

[95] Birney E, Stamatoyannopoulos JA, Dutta A, Guigo R, Gingeras TR, et al (2007) Identification and analysis of functional elements in 1% of the human genome by the ENCODE pilot project. Nature 447: 799–816. 17571346 10.1038/nature05874 PMC2212820

[96] Quinlan AR, Hall IM (2010) BEDTools: a flexible suite of utilities for comparing genomic features. Bioinformatics 26: 841–842. 20110278 10.1093/bioinformatics/btq033 PMC2832824

[97] Ben-Hur A, Weston J (2010) A user's guide to support vector machines. Methods in molecular biology 609: 223–239. 20221922 10.1007/978-1-60327-241-4_13

[98] Sonnenburg S, Ratsch G, Henschel S, Widmer C, Behr J, et al (2010) The SHOGUN Machine Learning Toolbox. J Mach Learn Res 99: 1799–1802.

[99] Salzberg S (1997) On Comparing Classifiers: Pitfalls to Avoid and a Recommended Approach. Data Mining and Knowledge Discovery 1: 317–327.

[100] Dietterich TG (1998) Approximate statistical tests for comparing supervised classification learning algorithms. Neural Comput 10: 1895–1923. 9744903 10.1162/089976698300017197

[101] Su AI, Wiltshire T, Batalov S, Lapp H, Ching KA, et al (2004) A gene atlas of the mouse and human protein-encoding transcriptomes. Proceedings of the National Academy of Sciences of the United States of America 101: 6062–6067. 15075390 10.1073/pnas.0400782101 PMC395923

[102] McLean CY, Bristor D, Hiller M, Clarke SL, Schaar BT, et al (2010) GREAT improves functional interpretation of cis-regulatory regions. Nature biotechnology 28: 495–501. 10.1038/nbt.1630 PMC4840234 20436461

[103] Grant CE, Bailey TL, Noble WS (2011) FIMO: scanning for occurrences of a given motif. Bioinformatics 27: 1017–1018. 21330290 10.1093/bioinformatics/btr064 PMC3065696

[104] Li Q, Ritter D, Yang N, Dong Z, Li H, et al (2010) A systematic approach to identify functional motifs within vertebrate developmental enhancers. Developmental biology 337: 484–495. 19850031 10.1016/j.ydbio.2009.10.019 PMC3330829

[105] Oksenberg N, Stevison L, Wall JD, Ahituv N (2013) Function and regulation of AUTS2, a gene implicated in autism and human evolution. PLoS genetics 9: e1003221. 23349641 10.1371/journal.pgen.1003221 PMC3547868

