# Supplementary figures and images for: Integrating Diverse Datasets Improves Developmental Enhancer Prediction
Source: PLoS Comput Biol. 2014 Jun 26;10(6):e1003677. doi: 10.1371/journal.pcbi.1003677 (PMC4072507; doi:10.1371/journal.pcbi.1003677)

**(A)**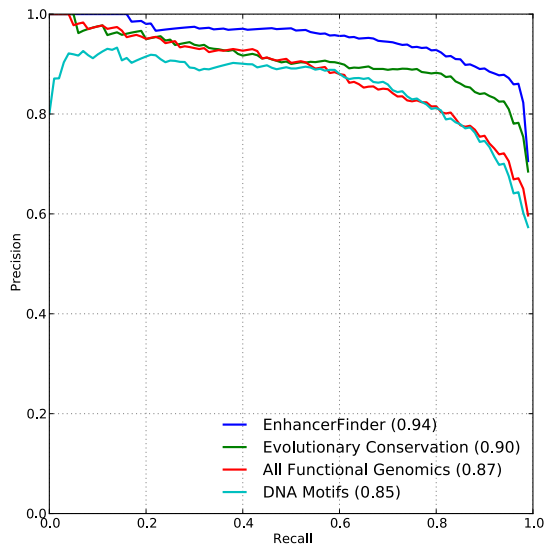**(B)**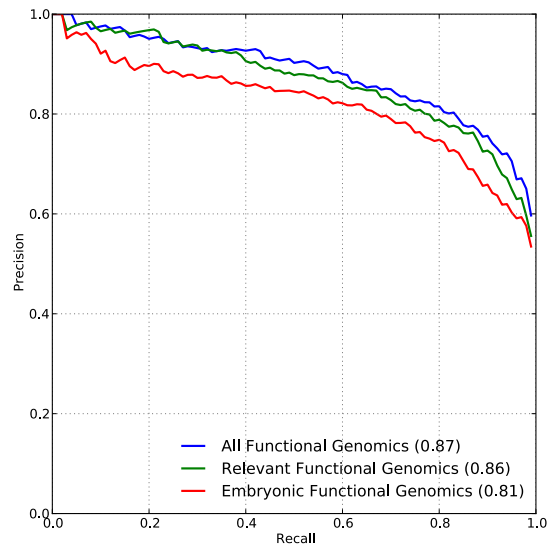**(C)**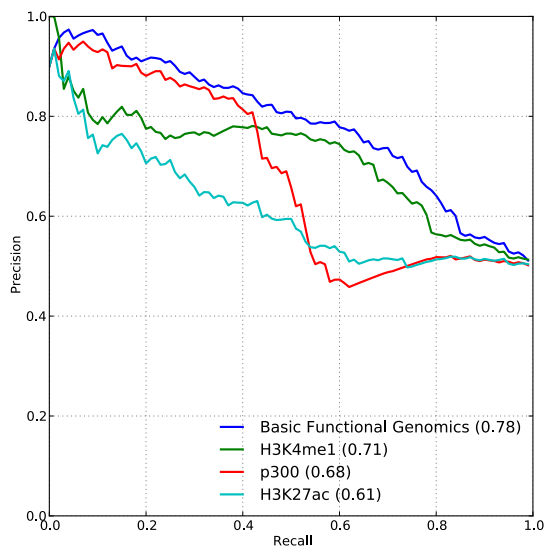**(D)**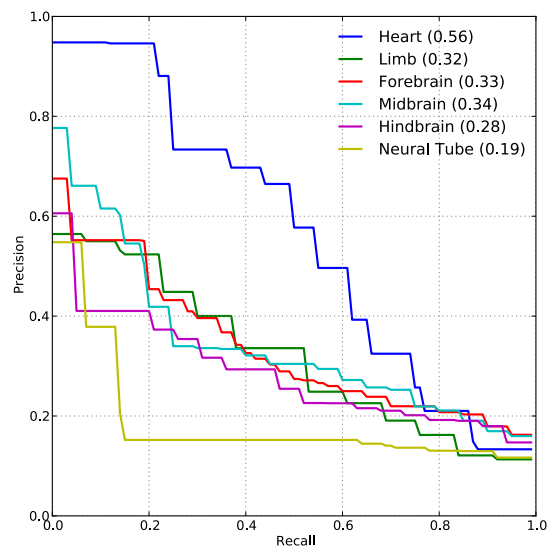

Supplement: Figure S1 — Precision-Recall curves corresponding to all ROC curves presented in the main text. (A) Figure 2A (B) Figure 3A (C) Figure 3B (D) Figure 4. A PR curve could not be created for Figure 2C, because we could not obtain the raw scores for regions from the CLARE web server. (PDF) [file pcbi.1003677.s001.pdf]

# Marks of 711 VISTA Positive Embryos

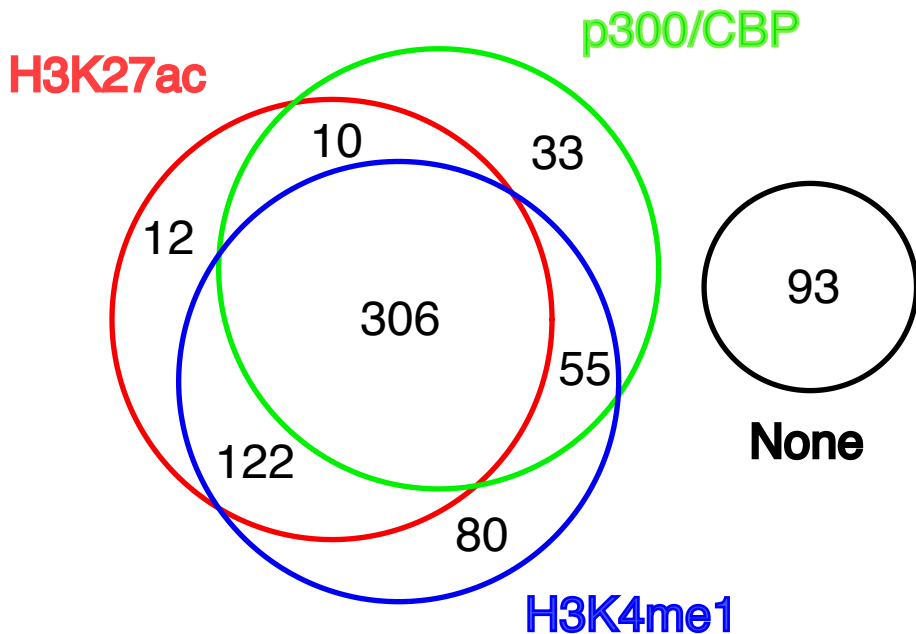

Supplement: Figure S2 — VISTA enhancers overlap many common marks of enhancers, but no common mark is universal to all VISTA enhancers. We computed the overlap between 711 VISTA enhancers and three common functional genomic marks of enhancers and found that 450 enhancers overlap H3K27ac (in any of 16 datasets from ENCODE), 563 overlap H3K4me1 (in any of 15 datasets from ENCODE), and 404 overlap p300/CBP (in any of 35 datasets from ENCODE and human tissues). Fewer than half of the enhancers (306) overlap all three common marks of enhancers, and 93 do not overlap any of those three functional genomics marks. All but five of the VISTA enhancers overlap a conservation peak (phastCons 46-way placental mammal). Four of these non-conserved enhancers overlap all three functional genomics marks, and one non-conserved enhancer overlaps just H3K27ac and H3K4me1. (PDF) [file pcbi.1003677.s002.pdf]

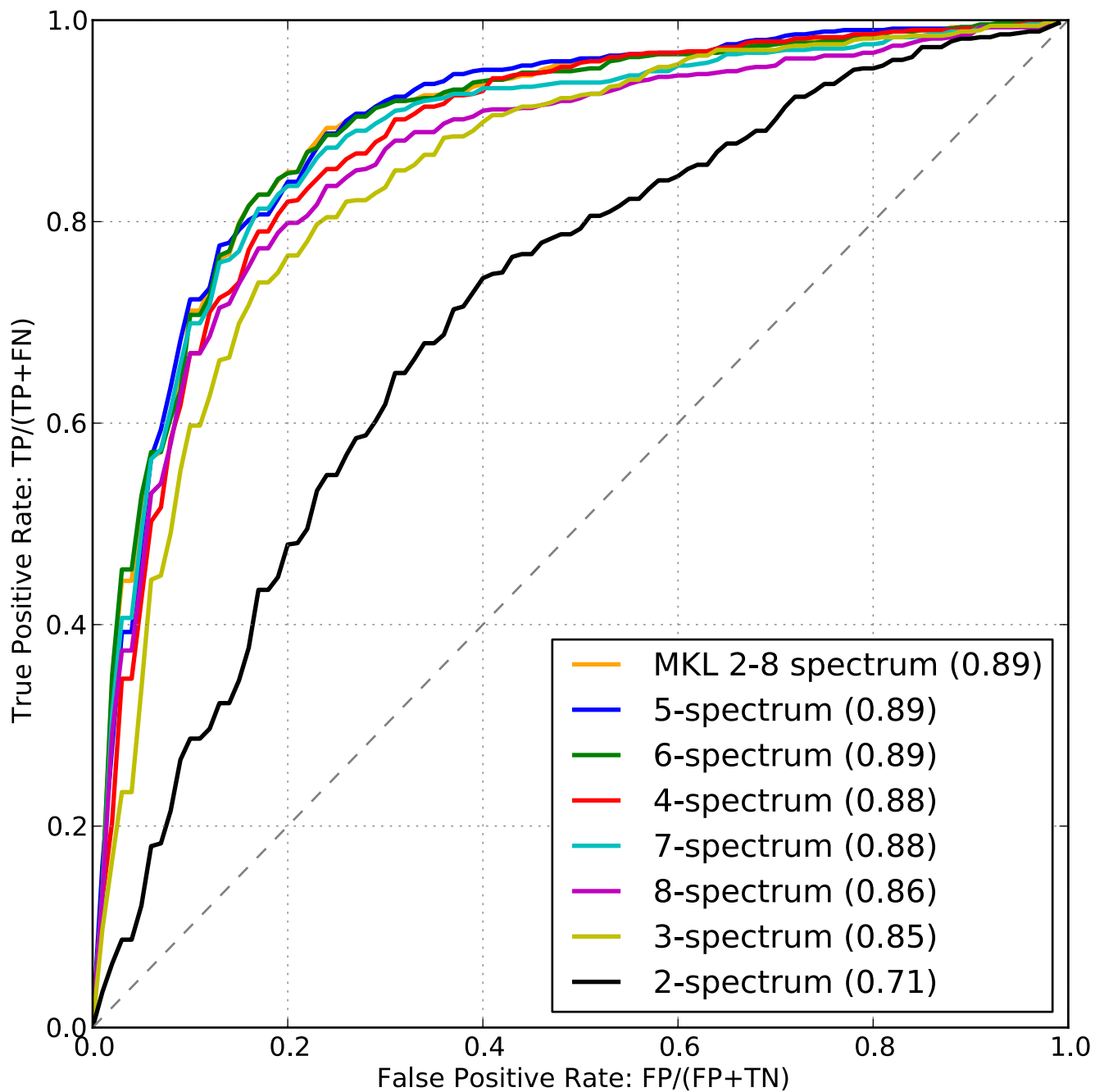

Supplement: Figure S3 — The 4-spectrum kernel performs competitively with other k-spectrum kernels and the combination of k-spectrum kernels. We analyzed the ability of spectrum kernels based on k-mer lengths between 2 and 8 to distinguish enhancers from the genomic background (Step 1). K-mers between 4 and 7 had the best performance. We also evaluated an MKL algorithm that combined each k-spectrum kernel, and it did not provide significant improvement over the best individual kernels. (PDF) [file pcbi.1003677.s003.pdf]

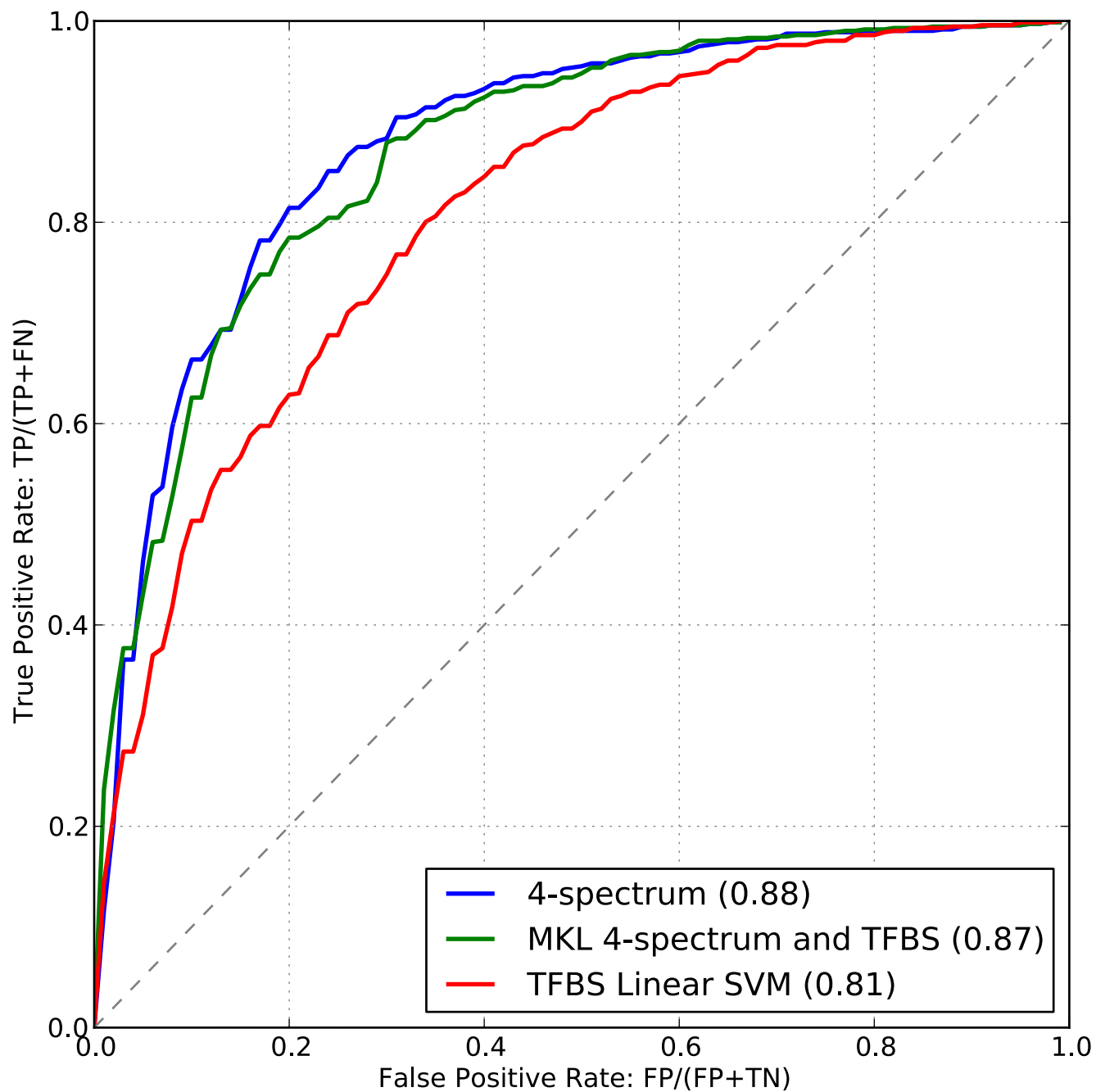

Supplement: Figure S4 — Considering known TFBS motifs does not improve the 4-spectrum kernel. Considering the number of occurrences of known TFBS motifs as features has recently been used in a linear SVM framework to predict enhancers [52]. To evaluate the utility of this approach, instead of and in addition to considering all k-mers, we created a linear SVM that used the number of hits to 1022 TF binding site matrices from TRANSFAC and JASPAR as computed by FIMO as features. That is the feature vector for each region consisted of 1022 elements, each of which was the number of significant hits for a different TF motif. This TFBS linear SVM (AUC = 0.81) did not perform as well as the 4-spectrum kernel (AUC = 0.88). We also evaluated an MKL algorithm that combined the 4-spectrum and TFBS kernels. This combined kernel did not perform any better than the 4-spectrum kernel suggesting that, at least under this encoding, TFBS motifs do not provide significant additional benefit in distinguishing enhancers from the genomic background. (PDF) [file pcbi.1003677.s004.pdf]

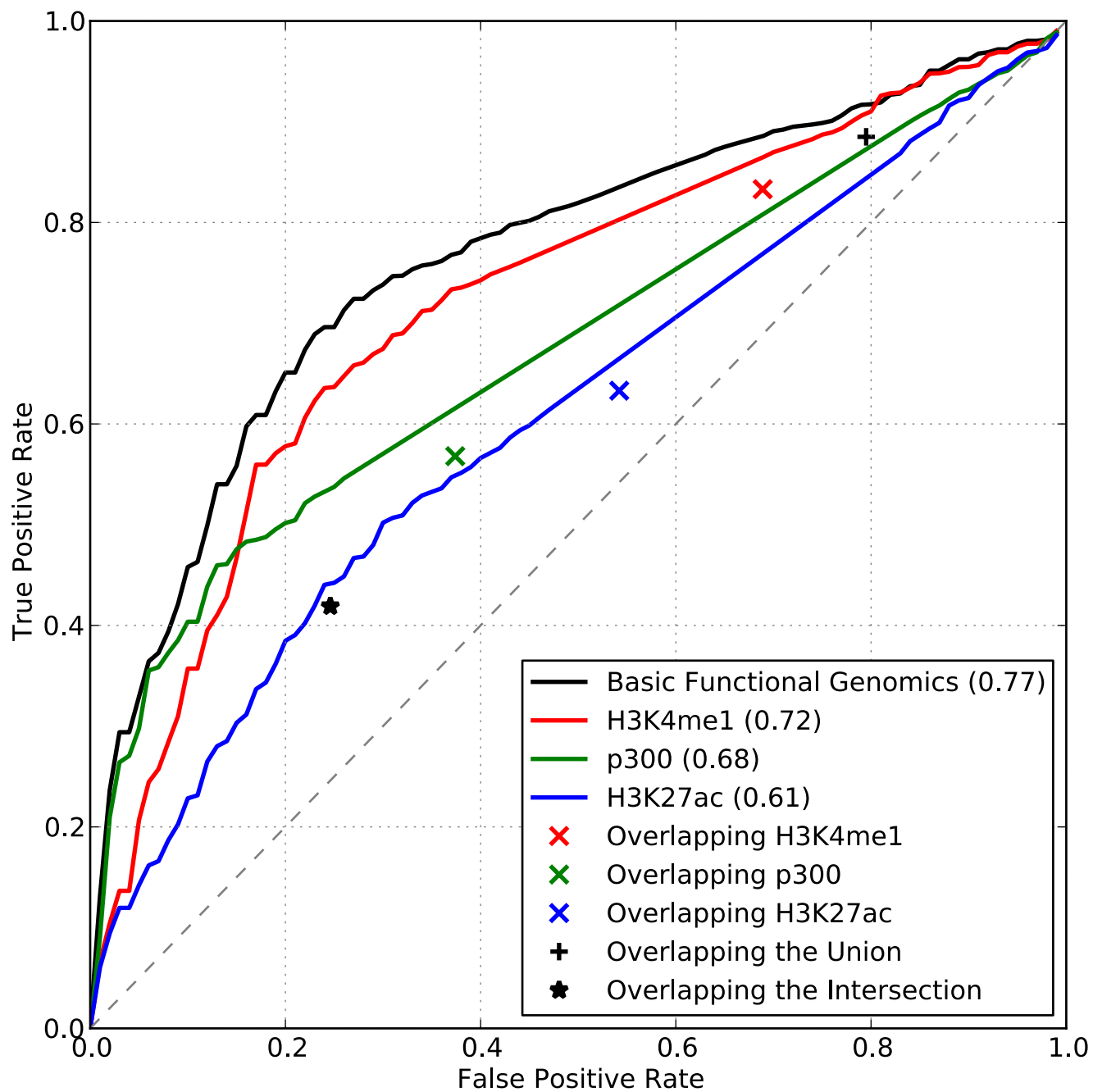

Supplement: Figure S5 — Combining functional genomics data with an SVM outperforms simply considering regions overlapping these data. The four solid lines shown are the same as in Figure 3B; they summarize the performance of these methods at distinguishing VISTA enhancers from the genomic background (Step 1). The X's give the performance of approaches that consider all regions overlapping a given feature as positives and all others as negatives. The + and * indicate the performance obtained by considering the union and intersection of H3K4me1, p300, and H3K27ac, respectively. For each feature, the linear SVM achieves better performance than simply considering all overlapping regions as positives. (PDF) [file pcbi.1003677.s005.pdf]

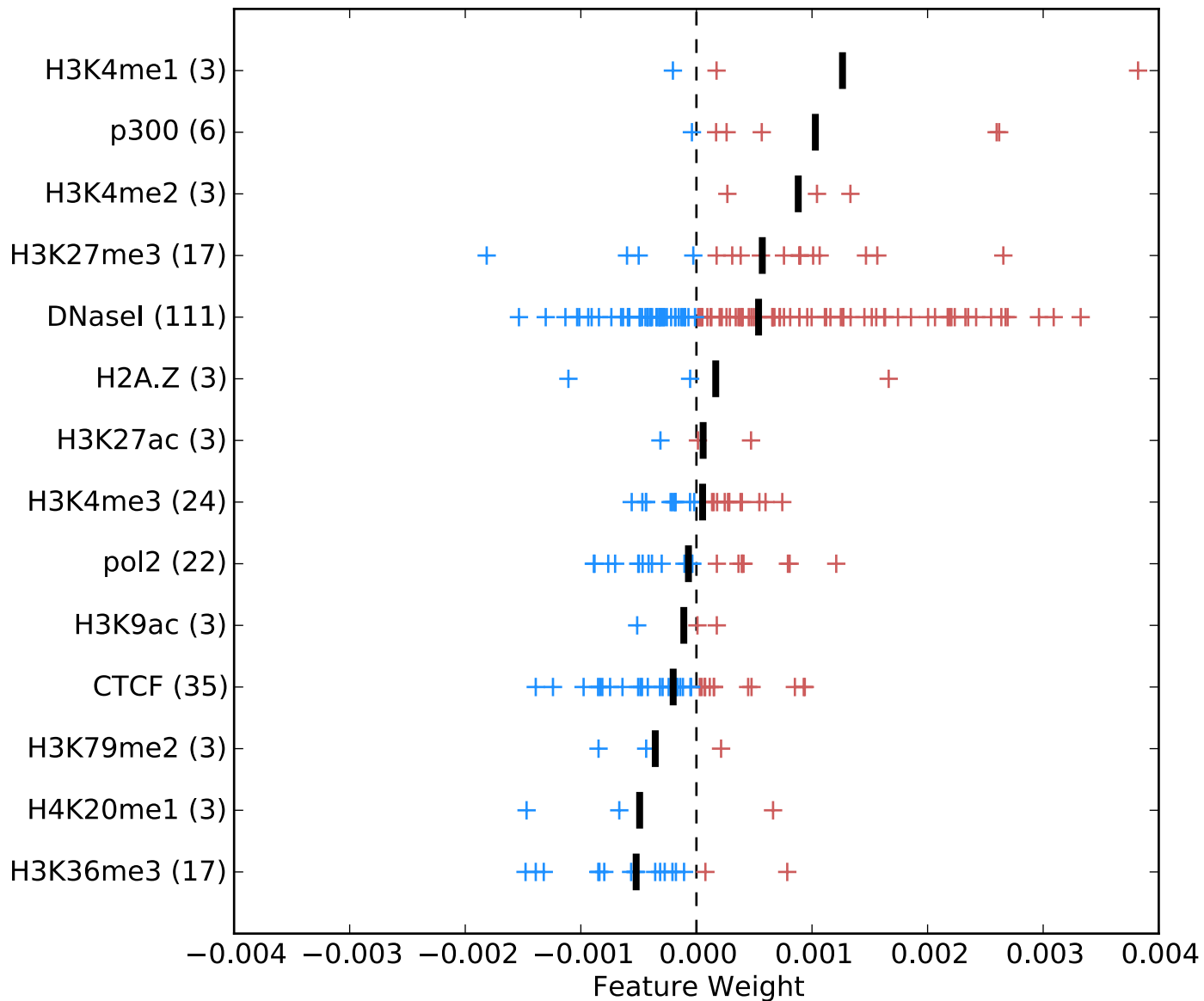

Supplement: Figure S6 — EnhancerFinder feature weights highlight the contribution of different functional genomics data types to enhancer predictions. Each “+” represents the contribution made by a single data feature, e.g. H3K4me1 peaks from embryonic stem cells, to the classification in EnhancerFinder Step 1 (developmental enhancers versus genomic background). Positive weights (red) indicate an association with enhancer activity in our analysis and negative weights (blue) suggest a lack of enhancer activity. The features plotted here come from a range of likely relevant contexts (Relevant Functional Genomics classifier; Table S1), and the number of data sets present for each feature type is given in parentheses. The black bar gives the average weight over all features of each type. In general, the features with high average weights, such as H3K3me1, p300, and H3K4me2, are known to be associated with enhancers, while those with large negative weights are associated with other types of genomic regions. However, no data type has uniformly positive or negative weights in all contexts. (PDF) [file pcbi.1003677.s006.pdf]

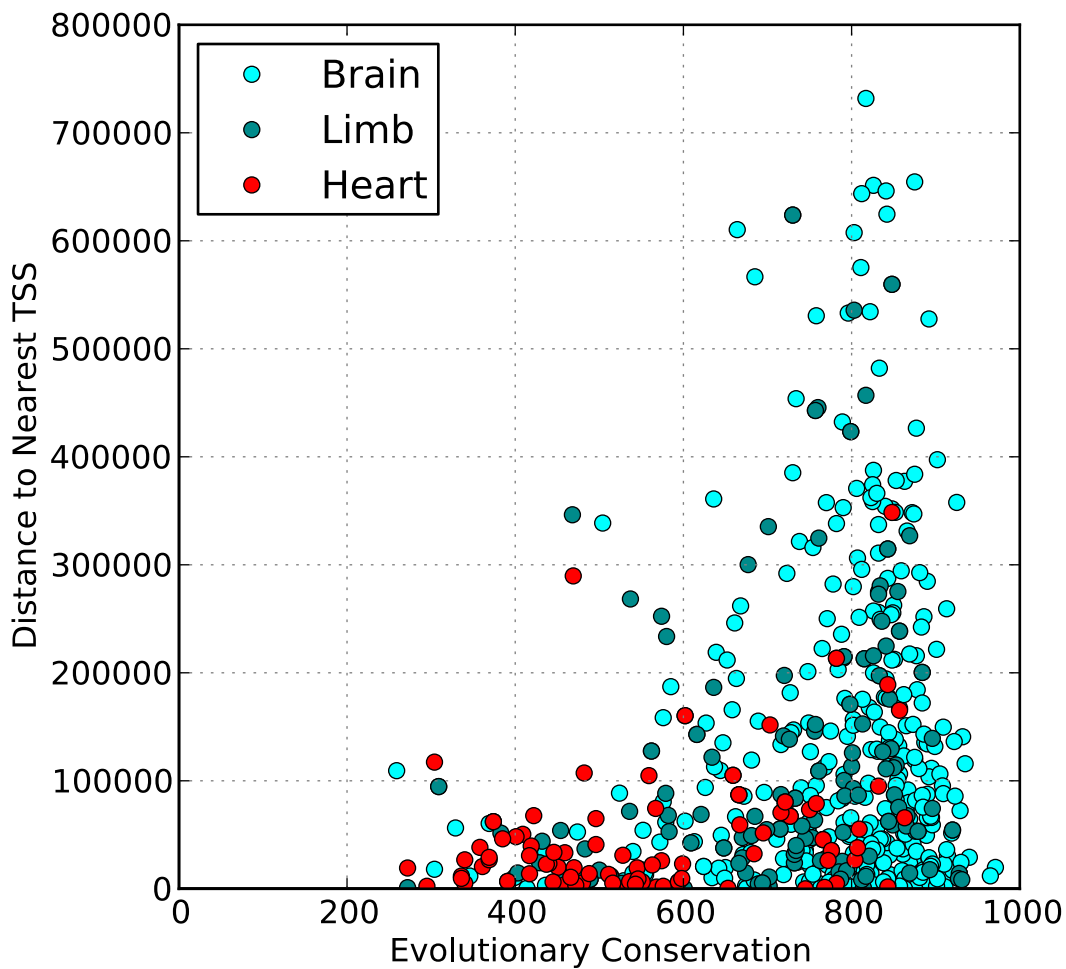

Supplement: Figure S7 — Heart enhancers are less conserved and closer to the nearest transcription start site (TSS) than limb and brain enhancers. Considering only limb and brain enhancers that are less evolutionarily conserved and close to a TSS improved our ability to identify them, but they are still more difficult to identify than heart enhancers. In addition to these features, heart enhancers have uniquely high GC content compared to other enhancers and the genomic background (Figure S7). (PDF) [file pcbi.1003677.s007.pdf]

**A**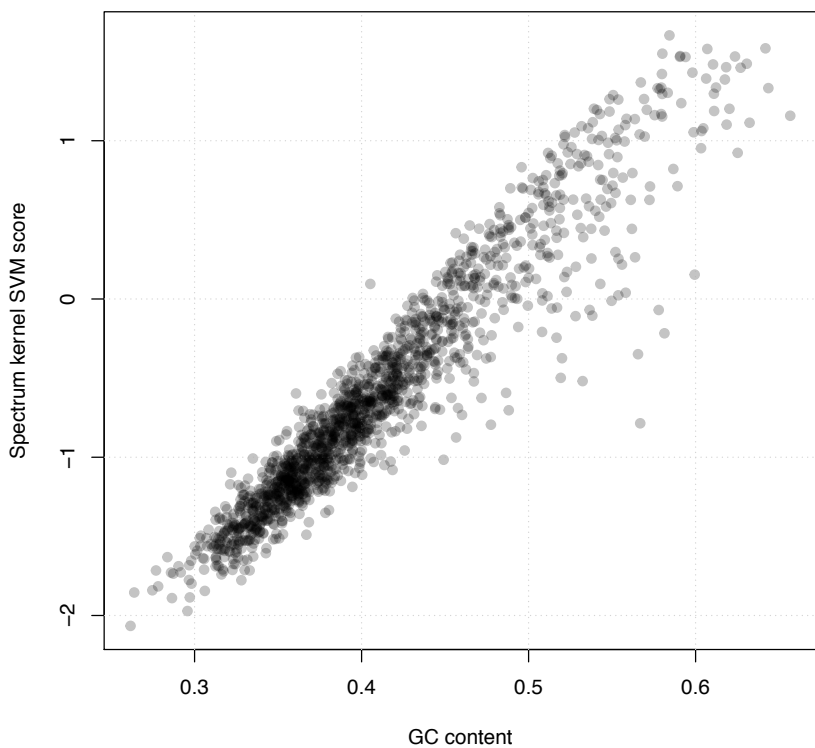**B**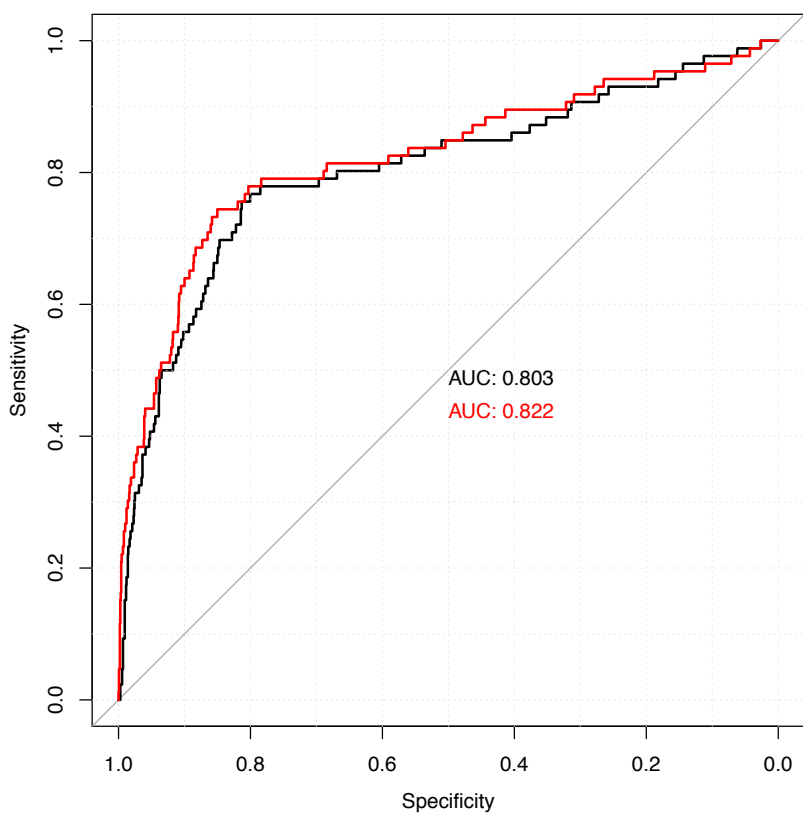

Supplement: Figure S8 — The uniquely high GC content of heart enhancers in VISTA enables accurate classification. The VISTA heart enhancers have higher GC content (49%) than other types of enhancers and the genomic background (∼40%). (A) The classification score from a spectrum kernel classifier trained to distinguish heart enhancers within VISTA (Step 2) is strongly correlated (Pearson rho = 0.95) with the GC content of the input region. (B) A classification algorithm based solely on GC content (black) performs competitively with the spectrum kernel (AUC of 0.80 vs. 0.82), and nearly as well as EnhancerFinder (0.85; Figure 4). (PDF) [file pcbi.1003677.s008.pdf]

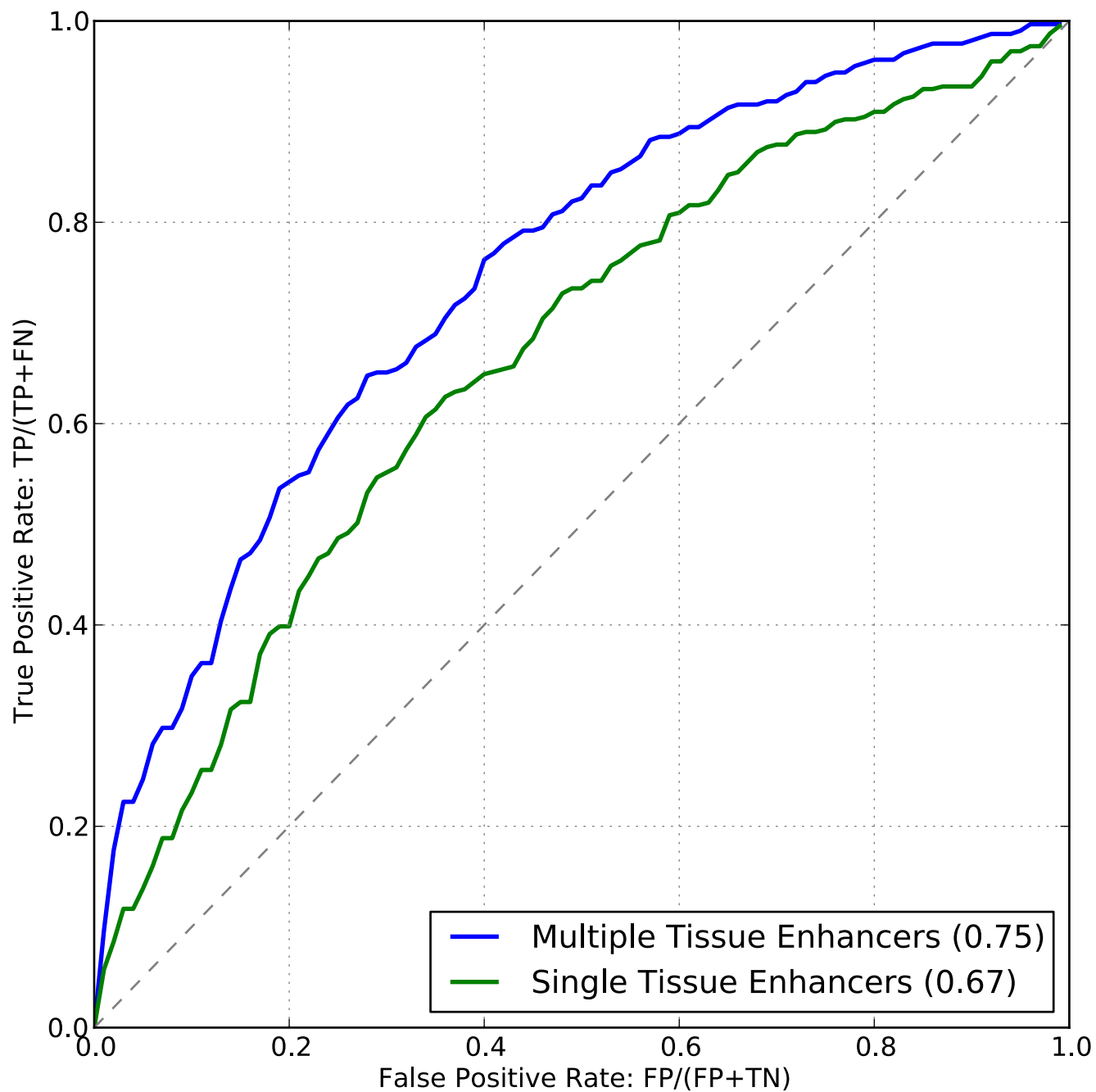

Supplement: Figure S9 — Enhancers active in multiple tissues are easier to identify than those active in a single tissue. There are 399 enhancers active in a single tissue at E11.5 in the VISTA database and 312 active in multiple tissues. EnhancerFinder is better able to distinguish the enhancers active in multiple tissues from the VISTA negatives (AUC = 0.75) than it is to distinguish single tissue enhancers from the negatives (AUC = 0.67). This trend also holds across each tissue individually. However, both sets are easy to distinguish from the genomic background (AUC = 0.96 for both, not shown). (PDF) [file pcbi.1003677.s009.pdf]

Figure S10

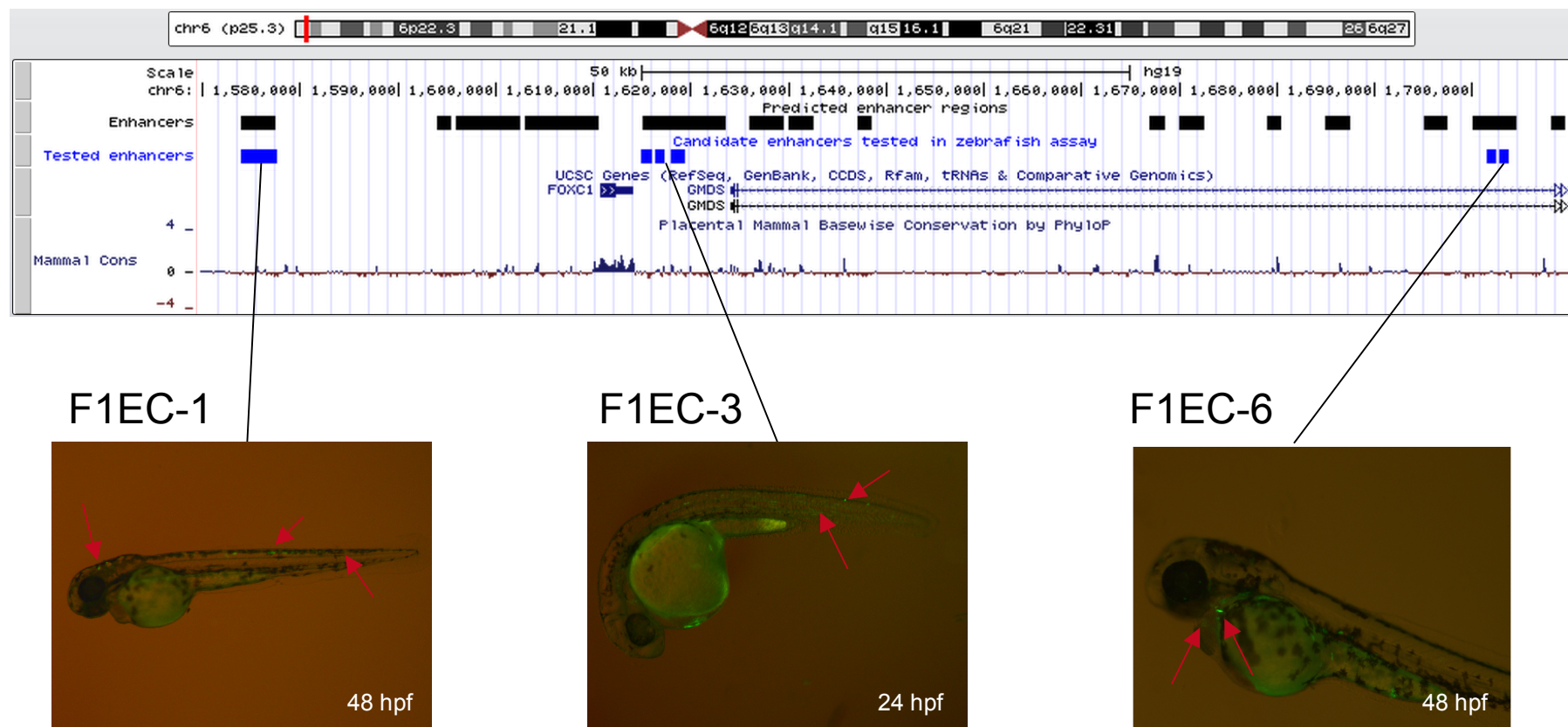

Supplement: Figure S10 — Three novel developmental enhancers near FOXC1 . This UCSC Genome Browser screenshot shows six candidate enhancer regions tested in transgenic zebrafish. Three of the regions showed positive or suggestive expression at 24 or 48 hpf. F1EC-1 drives expression at 48 hpf; the arrows highlight reproducible midbrain, spinal cord, and epidermis expression. F1EC-3 shows suggestive expression at 24 hpf in somitic muscles and the epidermis (arrows). F1EC-6 drives expression at 48 hpf in the pericardium and heart (suggestive). The other three tested candidate enhancers without corresponding zebrafish images were negative in the enhancer assay. See Table S6 for full list of expressed tissues seen in each candidate enhancer. (PDF) [file pcbi.1003677.s010.pdf]

**2xHAR.240**

Human:

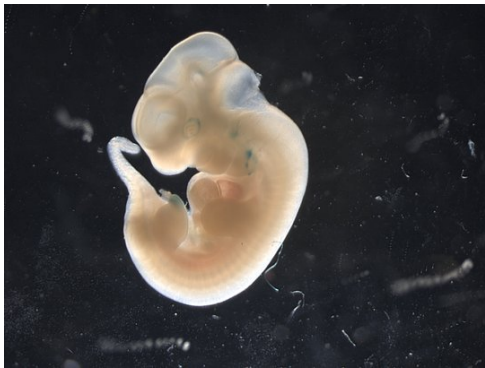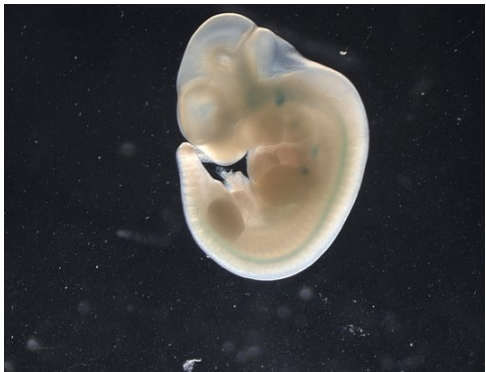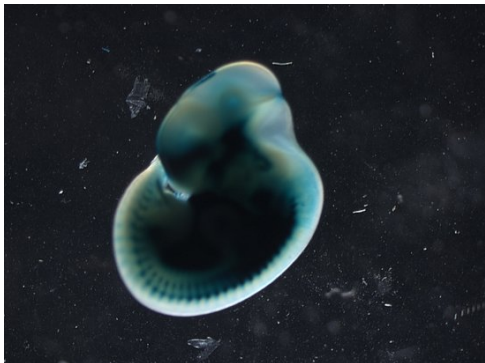

Chimp:

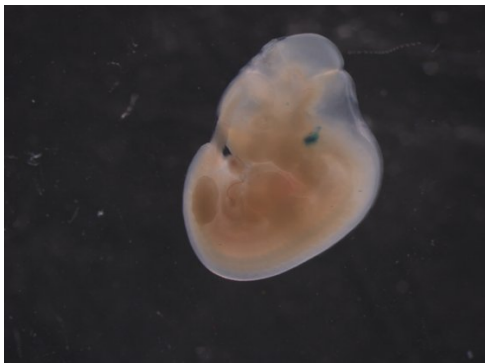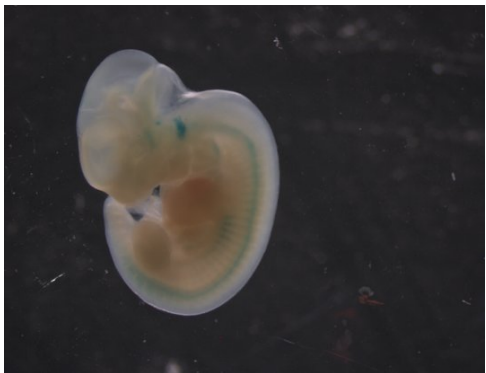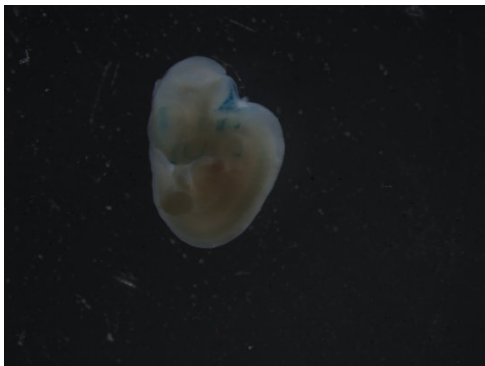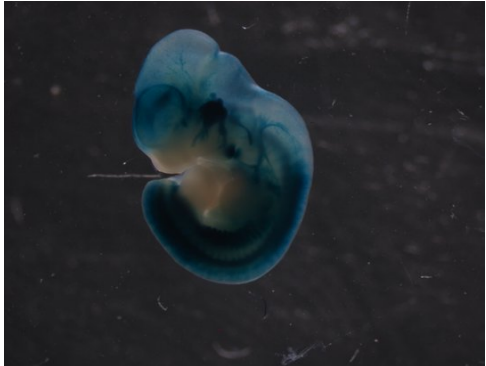

Supplement: Figure S11 — Transient transgenic mouse embryos support a novel cranial nerve enhancer near ZEB2 . Seven transient transgenic mouse embryos showed LacZ expression at embryonic day 11.5. Constructs containing a 999 bp region (hg19.chr2:145,234,541–145,235,539) including 2xHAR.240 near ZEB2, a minimal promoter, and LacZ were used for human. The orthologous region was used in the chimp construct (panTro2.chr2b:148,811,929–148,812,929). Three embryos with constructs containing the human version of the region of interest and four embryos containing the chimp sequence had staining. In all embryos, there was consistent expression in the cranial nerve. There does not appear to be a significant difference in the activity driven by the human and chimp sequences at this time point. (PDF) [file pcbi.1003677.s011.pdf]
