# Supplementary material for: Integrating Diverse Datasets Improves Developmental Enhancer Prediction
Source: PLoS Comput Biol. 2014 Jun 26;10(6):e1003677. doi: 10.1371/journal.pcbi.1003677 (PMC4072507; doi:10.1371/journal.pcbi.1003677)
Supplement: Table S2 — Genes near brain enhancers have significantly higher gene expression in brain and neural tissues than genes near heart enhancers. Brain- or heart-related tissues with significantly higher mean expression in genes associated with predicted brain enhancers compared to predicted heart enhancers. (DOC) [file pcbi.1003677.s013.doc]

**Table S2. Genes near brain enhancers have significantly higher gene expression in brain and neural tissues than genes near heart enhancers.** Brain- or heart-related tissues with significantly higher mean expression in genes associated with predicted brain enhancers compared to predicted heart enhancers.

| **Tissue** | **p-value** |
| --- | --- |
| Superior cervical ganglion | 8.85E-101 |
| Subthalamic nuclei | 1.28E-82 |
| Pons | 6.21E-77 |
| Medulla oblongata | 2.97E-65 |
| Globus pallidus | 1.16E-64 |
| Fetal brain | 4.72E-63 |
| Parietal lobe | 4.13E-62 |
| Trigeminal ganglion | 3.07E-60 |
| Dorsal root ganglion | 3.40E-57 |
| Atrioventricular node | 2.80E-56 |
| Occipital lobe | 4.24E-52 |
| Ciliary ganglion | 2.12E-45 |
| Temporal lobe | 2.08E-44 |
| Cingulate cortex | 3.47E-38 |
| Amygdala | 5.23E-29 |
| Whole brain | 1.69E-17 |
| Cerebellum | 3.70E-16 |
| Caudate nuclei | 1.27E-14 |
| Prefrontal cortex | 8.70E-14 |
| Hypothalamus | 4.04E-12 |
| Cerebellar peduncles | 5.63E-08 |
| Pituitary gland | 4.51E-05 |
| Thalamus | 0.039211 |
