# Supplementary material for: Integrating Diverse Datasets Improves Developmental Enhancer Prediction
Source: PLoS Comput Biol. 2014 Jun 26;10(6):e1003677. doi: 10.1371/journal.pcbi.1003677 (PMC4072507; doi:10.1371/journal.pcbi.1003677)
Supplement: Table S3 — Genes near heart enhancers have significantly higher gene expression in cardiac-related tissues than genes near brain enhancers. Brain- or heart-related tissues with significantly higher mean expression in genes associated with predicted heart enhancers compared to predicted brain enhancers. (DOC) [file pcbi.1003677.s014.doc]

**Table S3. Genes near heart enhancers have significantly higher gene expression in cardiac-related tissues than genes near brain enhancers.** Brain- or heart-related tissues with significantly higher mean expression in genes associated with predicted heart enhancers compared to predicted brain enhancers.

| **Tissue** | **p-value** |
| --- | --- |
| Cardiac myocytes | 2.56E-89 |
| Heart | 1.06E-55 |
| Whole blood | 8.94E-36 |
