# Supplementary material for: Integrating Diverse Datasets Improves Developmental Enhancer Prediction
Source: PLoS Comput Biol. 2014 Jun 26;10(6):e1003677. doi: 10.1371/journal.pcbi.1003677 (PMC4072507; doi:10.1371/journal.pcbi.1003677)
Supplement: Table S4 — The top 25 transcription factors for which binding sites were most prevalent in brain, heart, and limb enhancers. (DOC) [file pcbi.1003677.s015.doc]

**Table S4. The top 25 transcription factors for which binding sites were most prevalent in brain, heart, and limb enhancers.**

| **Brain enhancers** | **Heart enhancers** | **Limb enhancers** |
| --- | --- | --- |
| E2F1 | HIC1 | PITX2 |
| AHRHIF | AP2 | BACH1 |
| E2F1DP1RB | LRF | BACH2 |
| E2F4DP1 | E2F1 | MEF2 |
| E2F1DP1 | AP2GAMMA | CDX |
| E2F1DP2 | AP2ALPHA | NKX3A |
| E2F4DP2 | AHRHIF | AP1 |
| SP1 | SREBP2 | HMGIY |
| KROX | HIF1 | TEF |
| WT1 | LFA1 | OCT1 |
| HIC1 | EGR | POU3F2 |
| EGR | EGR3 | CDC5 |
| MAZR | KROX | FREAC7 |
| CKROX | SREBP | NKX62 |
| EGR1 | NRF1 | TBP |
| NRF1 | SREBP1 | RSRFC4 |
| AP2 | NFKAPPAB50 | HNF1 |
| CDPCR1 | WT1 | FOXO3A |
| MZF1 | HES1 | PIT1 |
| MAZ | AHRARNT | FOXJ2 |
| WHN | CREBP1 | FOXO3 |
| NRSE | SP1 | FOX |
| AHRARNT | MAZR | FOXD3 |
| GATA2 | EGR1 | NKX61 |
| OCT | CKROX | LHX3 |
