# Supplementary material for: Integrating Diverse Datasets Improves Developmental Enhancer Prediction
Source: PLoS Comput Biol. 2014 Jun 26;10(6):e1003677. doi: 10.1371/journal.pcbi.1003677 (PMC4072507; doi:10.1371/journal.pcbi.1003677)
Supplement: Table S6 — Candidate enhancer regions tested in zebrafish. We tested 10 candidate enhancer regions in a transgenic zebrafish assay. This table lists the genomic coordinates (hg19) and expression patterns observed for each construct at 24 and 48 hpf. A representative fish is shown for each positive enhancer in (Figures 7 and S9). Candidate enhancers on chromosome 6 are near FOXC1, and those on chromosome 16 are near FOXC2. N is the number of zebrafish alive at the specified time point, and * indicates expression patterns that are “suggestive,” but below the 15% threshold we used for confirmed enhancers. (DOC) [file pcbi.1003677.s017.doc]

**Table S6.** **Candidate enhancers tested in transgenic zebrafish assay.** We tested 10 candidate enhancer regions in a transgenic zebrafish assay. A representative fish is shown for each positive enhancer in the accompanying figures (Figure 7 and Supplementary Figure S10). GFP expression indicates positive enhancer activity. Genomic coordinates are hg19. Candidate enhancers on chromosome 6 are near *FOXC1*, and those on chromosome 16 are near *FOXC2*. N is the number of zebrafish alive at the specified timepoint. * indicates expression falls below the 15% threshold of “positive enhancer” and that expression in these tissues is suggestive and not confirmed.

| **Name** | **Genomic location (hg19)** | **N at 24 hpf** | **N at**  **48 hpf** | **Positive expression**  **at 24hpf** | **Positive expression**  **at 48hpf** |
| --- | --- | --- | --- | --- | --- |
| F1EC-1 | chr6:1,573,927-1,577,560 | 58 | 51 | Forebrain (12%)*  Midbrain (10%)*  Epidermis (12%)* | Eye (31%)  Forebrain (39%)  Hindbrain (16%)  Midbrain (45%)  Spinal cord (59%)  Pharyngeal arches (16%)  Somitic muscles (10%)*  Epidermis (29%) |
| F1EC-2 | chr6:1,614,903-1,616,336 | 63 | 68 | *(negative)* | *(negative)* |
| F1EC-3 | chr6:1,616,321-1,617,337 | 148 | 142 | Somitic muscles (10%)*  Epidermis (14%)* | *(negative)* |
| F1EC-4 | chr6:1,617,946-1,619,425 | 80 | 79 | *(negative)* | *(negative)* |
| F1EC-5 | chr6:1,701,475-1,702,475 | 142 | 137 | *(negative)* | *(negative)* |
| F1EC-6 | chr6:1,702,410-1,703,764 | 100 | 99 | Spinal cord (17%)  Yolk (11%)*  Epidermis (22%)  Pericardium (21%) | Spinal cord (11%)*  Yolk (30%)  Epidermis (37%)  Pericardium (23%)  Heart (12%)* |
| F2EC-1 | chr16:86,597,061-86,598,447 | 66 | 58 | Pericardium (20%) | Eye (28%)  Spinal cord (17%)  Somitic muscles (18%)  Caudal fin (14%)*  Epidermis (48%)  Pericardium (12%)* |
| F2EC-2 | chr16:86,602,685-86,605,098 | 59 | 59 | Forebrain (59%)  Hindbrain (56%)  Midbrain (61%)  Nerves (22%)  Notochord (14%)*  Spinal cord (66%)  Somatic muscles (41%)  Epidermis (44%)  Pericardium (37%)  Pre-organ region (24%) | Eye (10%)*  Forebrain (46%)  Hindbrain (14%)*  Midbrain (73%)  Nerves (62%)  Spinal cord (27%)  Somitic muscles (28%)  Epidermis (31%)  Pericardium (27%)  Heart (51%)  Organs - undetermined (19%) |
| F2EC-3 | chr16:86,605,112-86,606,111 | 100 | 100 | Eye (16%)  Forebrain (25%)  Midbrain (17%)  Spinal cord (13%)*  Somitic muscles (14%)*  Epidermis (24%)  Pericardium (16%) | Eye (17%)  Forebrain (16%)  Spinal cord (18%)  Somitic muscles (27%)  Yolk (16%)  Epidermis (40%)  Pericardium (17%)  Heart (23%) |
| F2EC-4 | chr16:86,606,310-86,607,263 | 70 | 70 | Midbrain (13%)*  Notochord (36%)  Spinal cord (14%)*  Somitic muscles (19%)  Yolk (24%)  Epidermis (60%)  Pericardium (16%) | Eye (13%)*  Notochord (34%)  Spinal cord (30%)  Somitic muscles (14%)*  Yolk (26%)  Epidermis (63%)  Pericardium (10%)*  Heart (21%) |
