# Supplementary material for: Integrating Diverse Datasets Improves Developmental Enhancer Prediction
Source: PLoS Comput Biol. 2014 Jun 26;10(6):e1003677. doi: 10.1371/journal.pcbi.1003677 (PMC4072507; doi:10.1371/journal.pcbi.1003677)
Supplement: Text S1 — Text describing additional analyses in support of the manuscript. (DOC) [file pcbi.1003677.s019.doc]

**SUPPLEMENTARY TEXT S1**

**The weights learned by EnhancerFinder to combine classifiers reflect the relative contribution of different data types to the enhancer predictions**

In addition to interpreting the performance of classifiers trained on different subsets of all the data considered by EnhancerFinder (Figures 2 and 3), we analyzed the weights learned by EnhancerFinder to combine each of the constituent classifiers to produce a single score (Methods). These classifier weights highlight the contribution of each feature type to the enhancer predictions. The weights learned for the feature sets give a qualitatively different view of the relationships than those described above from comparing the performance of classifiers trained on each feature set alone. The functional genomics classifier gets the highest weight (69%); the sequence motifs get the lowest (9%); and evolutionary conservation (22%) is in between. The difference in the weights of the functional genomics and sequence motif classifiers, despite their very similar performance, illustrates that the learned weights should not be interpreted as a proxy for performance. Rather, they indicate the importance of each feature set within the full trained EnhancerFinder classifier and are influenced by the utility of the features in the context of the other data sources considered.

**Classifier feature weight analysis**

Within the feature sets, the weight of an individual feature ranks its contribution to the prediction after accounting for the other data sets in the classifier (i.e., its adjusted, not marginal, effect). We explored the feature weights for individual functional genomics datasets. The weights for different datasets (i.e., different cell types or developmental time points) vary significantly within each assay, even when we considered only cellular contexts that are relevant to embryonic enhancer activity (see the definition of the **Relevant Functional Genomics** classifier) (Figure S5). Marks of accessible chromatin, like DNaseI hypersensitivity, and known enhancer associated features like p300, H3K4me1, H3K4me2, and H3K27ac received positive weights on average. The H3K27me3 mark also received generally positive weights; this is consistent with its role as a mark of “poised” enhancers in embryonic stem cells (ESCs) (from which many of the data sets shown in Figure S5 are derived) and association with genes active in embryogenesis . The H2A.Z histone variant has recently been associated with active regulatory regions, though it is not thought to be specific to enhancers . Consistent with this general role, we observed a range of weights and an average weight close to zero. Our algorithm gives overall negative weights to features such as RNA polymerase II and CTCF binding sites, and histone marks like H3K79me2, H4K20me1, and H3K36me3, all of which are known to be associated with other non-enhancer elements of the genome . However, the wide range of both positive and negative weights given to all classes of data suggests complex, context-dependent relationships with enhancer activity.

**Predicted enhancers are associated with relevant genomic regions**

To characterize and further validate our genome-wide enhancer predictions, we examined their genomic distribution with respect to several independent indicators of function, including the expression patterns and Gene Ontology (GO) annotations of nearby genes, hits to TFBS models, and single nucleotide polymorphisms (SNPs) from genome-wide association studies (GWAS).

*Genes near tissue-specific enhancers are enriched for expression in the relevant tissue.* We investigated expression patterns of the potential gene targets for our predicted tissue-specific enhancers. Using gene expression data from 79 human tissues from the GNF Gene Expression Atlas 2 , we compared mean expression levels of genes associated with brain versus heart enhancers by associating each predicted enhancer with the nearest TSS (See Methods). The GNF Atlas does not include enough relevant tissues to consider limb enhancers in this analysis. Genes near predicted brain enhancers had significantly higher expression levels in the 22 brain and neural tissues than genes associated with predicted heart enhancers (Table S2). Superior cervical ganglion, subthalamic nucleus, and pons show the most significantly elevated expression (all t-test p<0.001). Conversely, genes associated with heart enhancers showed elevated expression, compared to those associated with brain enhancers, in three of the four cardiovascular-related tissues: cardiac myocytes, heart, and whole blood (Table S3; all t-test p<0.001). Interestingly, expression in the atrioventricular node, which is part of the heart’s electrical conduction system, is higher for genes associated with predicted brain enhancers compared to predicted heart enhancers.

*Genes near tissue-specific enhancers are enriched for relevant functional annotations.* To explore functional annotations associated with genomic regions near predicted enhancers, we conducted GREAT analyses using the “basal plus extension” method to map annotations onto our predicted brain, heart, and limb enhancers. Gene Ontology (GO) Biological Process enrichment results suggest that our predicted developmental enhancers target genes that function in each of their relevant cell types and tissues (Figure 7). For example, TGF beta–receptor signaling, artery development, and artery morphogenesis are enriched among our heart enhancer predictions, while predicted brain enhancers are enriched for midbrain development and neuron differentiation. Predicted limb enhancers are enriched for kinase regulation and phosphorylation, which likely reflect the known role of kinases and phosphatases in limb patterning and development . Interestingly, some highly enriched GO Biological Process terms for predicted limb enhancers were also enriched for predicted heart enhancers (e.g., artery development and artery morphogenesis). This overlap appears to be due in part to the relatively large percentage of limb enhancer predictions that overlap heart enhancer predictions (e.g., the 1,960 overlapping enhancers comprise 25.6% of all limb enhancers and just 10.2% of all heart enhancers), and in part to the development of arteries and blood vessels in the limbs.

*Tissue-specific enhancers contain many relevant TFBS motifs.* We scanned the DNA sequences of our genome-wide tissue-specific enhancer predictions using TFBS motif models (see Methods). The most prevalent motifs (Table S4) differed between tissues and between enhancers predicted to be active only in a single tissue compared to all predicted enhancers of that tissue (e.g., enhancers unique to heart vs. all heart enhancers), suggesting potentially different functions and regulatory mechanisms for these classes of enhancers. For example, brain enhancers contained many binding sites for *NRSE* (neural restrictive silencer element), a TF thought to act as both a suppressor and enhancer in neural cells , whereas heart and limb did not have many *NRSE* binding sites. Binding sites for *NFkappaB*, which regulates transcription in heart morphogenesis , were abundant in heart enhancers but not brain or limb enhancers. Limb enhancers contained many binding sites for *LHX3*, a LIM homeobox gene that regulates limb growth and three-dimensional patterning , while brain and heart enhancers did not. See Table S4 for the full list of enriched TFs.

*GWAS SNPs are enriched in predicted enhancers.* We intersected the 9,687 SNPs in NHGRI’s GWAS catalog with our predicted enhancers and found that our enhancers contain 676 GWAS SNPs (Table S5), significantly more than expected at random (permutation p < 0.001). Looking at the tissue-specific predictions, we found 209 GWAS SNPs in the predicted heart enhancers (p < 0.001), 68 in predicted brain enhancers (p = 0.330), and 47 in the limb enhancers (p = 0.265).

**SUPPLEMENTARY FIGURE LEGENDS**

**Figure S1. Precision-Recall curves corresponding to all ROC curves presented in the main text.** (A) Figure 2A (B) Figure 2C The CLARE method, which is included in main text Figure 2C, was not included in this corresponding figure because we could not obtain the raw scores from regions from the web server (C) Figure 3A (D) Figure 3B (E) Figure 4.

**Figure S2. The 4-spectrum kernel performs competitively with other k-spectrum kernels and the combination of k-spectrum kernels.** We analyzed the ability of spectrum kernels based on k-mer lengths between 2 and 8 to distinguish enhancers from the genomic background (Step 1). K-mers between 4 and 7 had the best performance. We also evaluated an MKL algorithm that combined each k-spectrum kernel, and it did not provide significant improvement over the best individual kernels.

**Figure S3. Considering known TFBS motifs does not improve the 4-spectrum kernel.** Considering the number of occurrences of known TFBS motifs as features has recently been used in a linear SVM framework to predict enhancers . To evaluate the utility of this approach, instead of and in addition to considering all k-mers, we created a linear SVM that used the number of hits to 1022 TF binding site matrices from TRANSFAC and JASPAR as computed by FIMO as features. That is the feature vector for each region consisted of 1022 elements, each of which was the number of significant hits for a different TF motif. This TFBS linear SVM (AUC=0.81) did not perform as well as the 4-spectrum kernel (AUC=0.88). We also evaluated an MKL algorithm that combined the 4-spectrum and TFBS kernels. This combined kernel did not perform any better than the 4-spectrum kernel suggesting that, at least under this encoding, TFBS motifs do not provide significant additional benefit in distinguishing enhancers from the genomic background.

**Figure S4. Combining functional genomics data with an SVM outperforms simply considering regions overlapping these data.** The four solid lines shown are the same as in Figure 4B; they summarize the performance of these methods at distinguishing VISTA enhancers from the genomic background (Step 1). The X’s give the performance of an approach that considers all regions overlapping a given feature as positives and all others as negatives. The + and * indicate the performance obtained by considering the union and intersection of H3K4me1, p300, and H3K27ac, respectively. For each feature, the linear SVM achieves better performance than simply considering all overlapping regions as positives.

**Figure S5. EnhancerFinder feature weights highlight the contribution of different functional genomics data types to enhancer predictions.** Each “+” represents the contribution made by a single data feature, e.g. H3K4me1 peaks from embryonic stem cells, to the classification in EnhancerFinder Step 1 (developmental enhancers versus genomic background). Positive weights (red) indicate an association with enhancer activity in our analysis and negative weights (blue) suggest a lack of enhancer activity. The features plotted here come from a range of likely relevant contexts (**Relevant Functional Genomics** classifier; Table S1), and the number of data sets present for each feature type is given in parentheses. The black bar gives the average weight over all features of each type. In general, the features with high average weights, such as H3K3me1, p300, and H3K4me2, are known to be associated with enhancers, while those with large negative weights are associated with other types of genomic regions. However, no data type has uniformly positive or negative weights in all contexts.

**Figure S6. Heart enhancers are less conserved and closer to the nearest transcription start site (TSS) than limb and brain enhancers.** Considering only limb and brain enhancers that are less evolutionarily conserved and close to a TSS improved our ability to identify them, but they are still more difficult to identify than heart enhancers. The high GC content of heart enhancers proved essential to the ease of predicting them (Figure S7).

**Figure S7. The uniquely high GC content of heart enhancers in VISTA enables accurate classification.** The VISTA heart enhancers have higher GC content (49%) than other types of enhancers and the genomic background (~40%). (A) The classification score from a spectrum kernel classifier trained to distinguish heart enhancers within VISTA (Step 2) is strongly correlated (Pearson rho=0.95) with the GC content of the input region. (B) A classification algorithm based solely on GC content (black) performs competitively with the spectrum kernel (AUC of 0.80 vs. 0.82), and nearly as well as EnhancerFinder (0.85; Figure 5).

**Figure S8. Enhancers active in multiple tissues are easier to identify than those active in a single tissue.** There are 399 enhancers active in a single tissue at E11.5 in the VISTA database and 312 active in multiple tissues. EnhancerFinder is better able to distinguish the enhancers active in multiple tissues from the VISTA negatives (AUC=0.75) than it is to distinguish single tissue enhancers from the negatives (AUC=0.67). This trend also holds across each tissue individually. However, both sets are easy to distinguish from the genomic background (AUC=0.96 for both, not shown).

**Figure S9. Transient transgenic mouse embryos support a novel cranial nerve enhancer near *ZEB2*.** Seven transient transgenic mouse embryos showed *LacZ* expression at embryonic day 11.5. Constructs containing a 999 bp region (hg19.chr2:145,234,541-145,235,539) including 2xHAR.240 near *ZEB2*, a minimal promoter, and *LacZ* were used for human. The orthologous region was used in the chimp construct (panTro2.chr2b:148,811,929-148,812,929). Three embryos with constructs containing the human version of the region of interest and four embryos containing the chimp sequence had staining. In all embryos, there is consistent expression in the cranial nerve. There does not appear to be a significant difference between human and chimp at this time point.

**SUPPLEMENTARY TABLE LEGENDS**

**Table S1. Functional genomics features used in our analysis.** This Excel spreadsheet lists the files used from ENCODE (http://genome.ucsc.edu/ENCODE/) or GEO (<http://www.ncbi.nlm.nih.gov/geo/>). There is a sheet for each of the classifiers based on functional genomics data that lists all data files used. ENCODE data set names are UCSC track names. GEO data set names are GEO identifiers.

**Table S2. Genes near brain enhancers have significantly higher gene expression in brain and neural tissues than genes near heart enhancers.** Brain- or heart-related tissues with significantly higher mean expression in genes associated with predicted brain enhancers compared to predicted heart enhancers.

**Table S3. Genes near heart enhancers have significantly higher gene expression in cardiac-related tissues than genes near brain enhancers.** Brain- or heart-related tissues with significantly higher mean expression in genes associated with predicted heart enhancers compared to predicted brain enhancers.

**Table S4. The top 25 transcription factors for which binding sites were most prevalent in brain, heart, and limb enhancers.**

**Table S5. 676 GWAS SNPs are found in predicted enhancers.**

This Excel spreadsheet lists all GWAS SNPs from the NHGRI database that fall within one of our predicted enhancers.

**DATA FILE S1**

This ZIP archive contains BED files (hg19 coordinates) with EnhancerFinder’s genome-wide enhancer predictions, along with the MKL scores, for general developmental enhancer activity, brain, heart, and limb enhancers. The general prediction file also lists the H3K27ac and H3K4me1 marks from the feature data overlapping each predicted enhancer.

References for Supplementary Text S1

1. Rada-Iglesias A, Bajpai R, Swigut T, Brugmann SA, Flynn RA, et al. (2011) A unique chromatin signature uncovers early developmental enhancers in humans. Nature 470: 279-283.

2. Jin C, Zang C, Wei G, Cui K, Peng W, et al. (2009) H3.3/H2A.Z double variant-containing nucleosomes mark 'nucleosome-free regions' of active promoters and other regulatory regions. Nature genetics 41: 941-945.

3. Zhou VW, Goren A, Bernstein BE (2011) Charting histone modifications and the functional organization of mammalian genomes. Nature reviews Genetics 12: 7-18.

4. Su AI, Wiltshire T, Batalov S, Lapp H, Ching KA, et al. (2004) A gene atlas of the mouse and human protein-encoding transcriptomes. Proceedings of the National Academy of Sciences of the United States of America 101: 6062-6067.

5. McLean CY, Bristor D, Hiller M, Clarke SL, Schaar BT, et al. (2010) GREAT improves functional interpretation of cis-regulatory regions. Nature biotechnology 28: 495-501.

6. Dudley AT, Tabin CJ (2003) Deconstructing phosphatases in limb development. Nature cell biology 5: 499-501.

7. Saxton TM, Ciruna BG, Holmyard D, Kulkarni S, Harpal K, et al. (2000) The SH2 tyrosine phosphatase shp2 is required for mammalian limb development. Nature genetics 24: 420-423.

8. Schoenherr CJ, Anderson DJ (1995) The neuron-restrictive silencer factor (NRSF): a coordinate repressor of multiple neuron-specific genes. Science 267: 1360-1363.

9. Hernandez-Gutierrez S, Garcia-Pelaez I, Zentella-Dehesa A, Ramos-Kuri M, Hernandez-Franco P, et al. (2006) NF-kappaB signaling blockade by Bay 11-7085 during early cardiac morphogenesis induces alterations of the outflow tract in chicken heart. Apoptosis : an international journal on programmed cell death 11: 1101-1109.

10. Tzchori I, Day TF, Carolan PJ, Zhao Y, Wassif CA, et al. (2009) LIM homeobox transcription factors integrate signaling events that control three-dimensional limb patterning and growth. Development 136: 1375-1385.

11. Hindorff LA, Sethupathy P, Junkins HA, Ramos EM, Mehta JP, et al. (2009) Potential etiologic and functional implications of genome-wide association loci for human diseases and traits. Proceedings of the National Academy of Sciences of the United States of America 106: 9362-9367.

12. Burzynski GM, Reed X, Taher L, Stine ZE, Matsui T, et al. (2012) Systematic elucidation and in vivo validation of sequences enriched in hindbrain transcriptional control. Genome research 22: 2278-2289.
